# qKAT: a high-throughput qPCR method for KIR gene copy number and haplotype determination

**DOI:** 10.1186/s13073-016-0358-0

**Published:** 2016-09-29

**Authors:** W. Jiang, C. Johnson, N. Simecek, M. R. López-Álvarez, D. Di, J. Trowsdale, J. A. Traherne

**Affiliations:** 1 Cambridge Institute for Medical Research, University of Cambridge, Cambridge Biomedical Campus, Wellcome Trust/MRC Building, Hills Road, Cambridge, CB2 0XY UK; 2 Immunology Division, Department of Pathology, University of Cambridge, Tennis Court Road, Cambridge, CB2 1QP UK

**Keywords:** Killer cell immunoglobulin-like receptor (*KIR*), Copy number variation, Haplotype, Real-time quantitative polymerase chain reaction

## Abstract

Killer cell immunoglobulin-like receptors (KIRs), expressed on natural killer cells and T cells, have considerable biomedical relevance playing significant roles in immunity, pregnancy and transplantation. The *KIR* locus is one of the most complex and polymorphic regions of the human genome. Extensive sequence homology and copy number variation makes *KIR*s technically laborious and expensive to type. To aid the investigation of *KIR*s in human disease we developed a high-throughput, multiplex real-time polymerase chain reaction method to determine gene copy number for each *KIR* locus. We used reference DNA samples to validate the accuracy and a cohort of 1698 individuals to evaluate capability for precise copy number discrimination. The method provides improved information and identifies *KIR* haplotype alterations that were not previously visible using other approaches.

**Electronic supplementary material:**

The online version of this article (doi:10.1186/s13073-016-0358-0) contains supplementary material, which is available to authorized users.

## Background

Complex and multi-allelic copy number variation (mCNV) is abundant in the human genome and is a potential source of genetic diversity in relation to disease [1]. Genes involved in immunity and defence seem to be especially prone to mCNV [2], presumably driven by selection pressure from pathogens. Human leukocyte antigen (HLA) DRB, complement component 4 (C4) loci in the MHC and leukocyte immunoglobulin-like receptor (LILR) loci are recognised examples of multicopy gene families with mCNV linked to disease susceptibility [3–7]. Another genomic region of interest in this regard encompasses the killer cell immunoglobulin-like receptor (KIR) genes [8, 9]. KIR were discovered nearly 20 years ago by serological methods [10, 11]. Subsequently they have been shown to have important physiological and biomedical relevance in wide-ranging conditions including pregnancy, infection, autoimmunity, cancer and transplantation [12]. *KIR* associations involve epistasis with their variable cognate HLA ligands. Complex interactions of unlinked loci like these may account for some of the heritability void left by genome-wide association studies (GWAS).

*KIR* genes, which are part of the leukocyte receptor complex (LRC) in human chromosomal region 19q13.4, have evolved rapidly in parallel with their HLA ligands through varying types of selection. As such, *KIR* genes exhibit high diversity in copy number and haplotypes. Unlike normal homologous recombination, chromosomal crossovers in the *KIR* cluster may misalign because the genes are closely arranged head-to-tail and they are homologous in sequence to one another. The process, known as non-allelic homologous recombination (NAHR), generates novel expanded and contracted haplotypes with duplication or deletion of whole genes (between ~11 and 18 kb in size), multiple genes and formation of novel fusion genes [3]. Gene dose effects at the mRNA and protein level have been seen for *KIR* genes, namely *KIR2DL2/L3* and *KIR3DS1*. KIR expression is stochastic and the number of NK cells expressing a given KIR correlates linearly with the total number of copies of the gene carried by the individual [13]. Thus, the overall responsiveness of the NK cell repertoire directly relates to *KIR* haplotype content. This has implications for NK cell-mediated alloreactivity in hematopoietic stem cell transplantation, where donors with a high proportion of alloreactive NK cells have higher levels of cytolytic activity against leukemic cells [14]. Furthermore, *KIR* copy number variation (CNV) has been shown to correlate with protection from certain viruses such as HCV and HIV [15, 16].

The current *KIR* typing techniques that employ specific primers (PCR-SSP) [17–19] or oligonucleotides (PCR-SSO) [20] have drawbacks when applied to large-scale studies of genetically complex diseases; they are time-consuming, expensive and labour-intensive. *KIR*s are refractory to high throughput methods because of extensive sequence homology, allelic and copy number variation. For this reason, *KIR* studies have been limited to date by their relatively small scale and they have been ignored in GWAS to date. In addition, recent studies indicate that structural variations in haplotypes have been overlooked. The conventional methods, such as PCR-SSP, PCR-SSO and MALDI-TOF [21] cannot detect such variation as they lack the ability to quantify gene number, instead providing only ‘presence/absence’ status for a gene.

In this paper, we describe a high-throughput method to determine copy number of each *KIR* locus, using quantitative polymerase chain reaction (PCR) with dual-labelled hydrolysis probes, which we have called qKAT for quantitative *KIR* semi-automated typing. This method can help simplify disease analysis by identifying unusual haplotypes so that the major haplotypes can be analysed separately. We extend the approach to *LILR* loci, demonstrating that the underlying strategy of qKAT offers a model for analysing and visualizing other highly variable mCNV regions.

In real-time PCR, the fluorescent threshold value (cycle of quantification, C_q_) correlates linearly with logarithmic value of starting DNA copy number [22]. This method can determine the quantity of target DNA sequence specifically and accurately, therefore it has been used extensively for gene quantification, especially in gene expression studies. Compared with complementary DNA quantification in gene expression studies, copy numbers of target gene derived from both chromosomes is slightly different. The relative DNA copy number measured against a reference gene is always an integer ratio. In addition, it is a very small change compared to gene expression (2× and 1.5× for 1–2 and 2–3 copy changes, respectively).

Multiplex quantitative PCR has the advantage of simultaneously amplifying several products in the same tube, using spectrally distinct fluorophores to detect each amplification. The method allows reduction of DNA requirements, reagent costs, human labour and time. Using internal controls increases the reliability of the results. The optimised multiplex assays considerably reduce the cost and setup time by high throughput. Well-to-well variation is minimised in multiplex PCR since target assay and reference assay are run in the same tube at the same time, providing extra confidence in the results.

## Methods

### Multiplex quantitative PCR assay

**Fig. 1:**
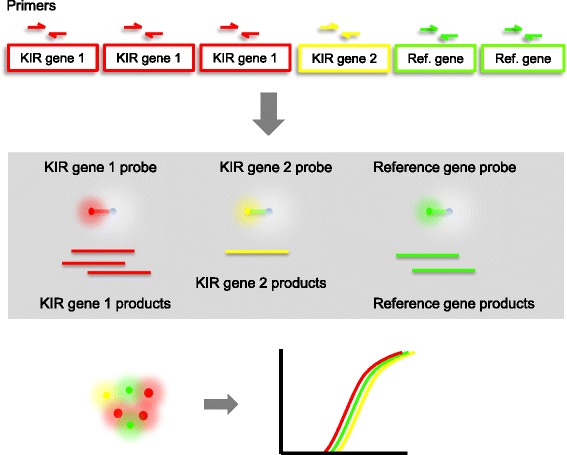
*Schematic view* of the *KIR* multiplex qPCR assay qKAT. Sequence specific primers are used for relative quantification of target *KIR* genes against a reference gene of fixed copy number. Each multiplex qPCR assay detects the simultaneous amplification of two target *KIR* genes and one reference gene. Two sets of primers target different exons of the two *KIR* genes and one pair of primers amplify the reference gene. Dual-labelled hydrolysis probes that are specific to each amplicon are used to monitor the PCR amplification in real-time

For *KIR* assays, ten multiplex quantitative PCR reactions were carried out in a triplex format that included three probes targeting three different amplicons. The optimisations of primer and probe concentrations are shown in Additional file 1: Figures S1 and S2. The overall performance of each reaction was tested using standard curves (see Additional file 1: Figure S3) and the PCR efficiencies of each reaction are given in Additional file 1: Table S1. Each multiplex reaction detects two *KIR* genes and one endogenous reference gene (*STAT6*) that is located on a different chromosome and always has two copies in a diploid genome [23] (Fig. 1) as corroborated using the Database of Genomic Variants (http://dgv.tcag.ca/dgv/app/). Altogether, copy numbers of 20 markers were ascertained for 17 *KIR* genes and their important variants (*2DL1–5*, *2DS1–3*, *2DS4* (separate assays for the gene, full-length variant [FL] and deletion variant [del]), *2DS5*, *3DL1–3*, *3DS1*, *2DP1* and *3DP1*). For *3DL1* and *3DL2*, two reactions were used to target different parts of the gene to identify known fusion genes [24]. *LILR* gene copy number was determined using duplex reactions including one *LILR* target and the reference gene (Additional file 1: Table S2).

The reaction mix containing genomic DNA, primers, probes, Taq polymerase and buffer was dispensed into 384-well plates. Five nanograms of each DNA sample were plated into 384-well plates with four replicates. Three control samples of known copy number were included in each run. Multiplex PCR reactions were performed on a Roche LightCycler 480 using absolute quantification settings. Fluorescent signals were collected at the end of each cycle for further analysis.

After PCR amplification, C_q_ values were calculated using either the Second Derivative Maximum Method or the Fit Points Method. The copy number was determined by relative quantification analysis using the comparative C_q_ method (also known as delta delta C_q_ method, ΔΔC_q_). This method compares the cycle of quantification between the test sample and a calibrator sample with known copy number. In cases where a copy number calibrator sample was not available, the copy number analysis could be performed using the most frequent copy number expected in the samples.

### Primer design

Based on PCR-SSP (PCR amplification with sequence-specific primers), our method used primers with 3' ends specifically matching a nucleotide which is unique to a given *KIR*/*LILR* gene. The gene-specific primers were designed to detect all the alleles of the given gene (Additional file 1: Table S3). In this assay, 21 primer pairs were used to detect all 16 *KIR* genes, important *KIR* gene variants and one reference gene. Primers were designed to produce amplicons in the range of 78–210 bp in size. Compared with the previous PCR-SSP and PCR-SSO methods, the short amplicons allowed the PCR reactions to achieve maximum efficiency. The Tm (melting temperature) of each primer was adjusted to between 48 °C to 57 °C, by adjusting the length of primers.

Initially, 37 primer pairs were designed for all 16 *KIR* genes and some variants. There was more than one reaction for most genes. Gel electrophoresis was used to check each PCR reaction using a known positive DNA sample and two negative DNA samples (for non-framework genes) and a non-template control. Any reactions with non-specific bands, weak amplification and strong primer-dimer were excluded. Due to the limited choice of unique nucleotides for *KIR*-specific priming, some primers were designed with GC content of up to 70.6 %.

### Probe design

Due to the high sequence similarity between *KIR*/*LILR* genes, it is difficult to design specific probes. Dual-labelled probes were designed to exons sequences that are conserved between more than one gene (Additional file 1: Table S4). The same probe could therefore be used in different multiplex assays. Generic probes were feasible because the specificity for each reaction is controlled by the primers and never the same region for different targets was amplified in the same multiplex assay. Using generic probes between different reactions greatly reduced the cost since probes account for a large part of reagent costs. The primer and probe sequences for qKAT are given in Additional file 1 to allow judgment of whether they are appropriate for defining the currently known polymorphisms and variants discovered in the future. Primer and probe combinations used in each reaction are given in Additional file 1: Table S5.

The fluorophores used in the multiplex assays were FAM, Dragonfly Orange and Cy5. The maximum emission wavelengths of the three dyes are: 518 nm, 576 nm and 667 nm, respectively. These three dyes have distant emission spectra, which minimise the fluorescence signal from one dye bleeding into adjacent channels (signal crosstalk). Non-fluorescent black-hole quenchers (BHQ) were used because they have advantages over the other quenchers such as TAMRA, since they absorb the excitation energy from fluorophore and convert it into heat rather than re-emit this energy as light with a different wavelength. This is useful for multiplex PCR reactions, since there is no emitted light from the quencher to interfere with the reporter fluorophores, resulting in less background signals and hence have better signal/background ratios.

### Determination DNA copy number using relative quantification analysis

*KIR* and *LILR* copy number of genomic DNA sample was determined using comparative C_q_ (ΔΔC_q_) relative quantification analysis [25–28]. The relationship between calculated copy number without efficiency correction and true copy number is illustrated in Additional file 1: Figure S4. Calculated copy number without efficiency correction and true copy number diverge significantly as the ΔC_q_ in reference assay increases. This explains that apart from PCR efficiency, the ΔC_q_ in reference assay could affect the copy number calculation. The ΔC_q_ in the reference assay actually reflects the difference of DNA concentrations between sample and calibrator. If the DNA concentration is well quantified and controlled in a reasonable range, then the copy number calculation will not be affected much even without PCR efficiency correction. In addition, the parameters from our assays are slightly better than the assumed values. For example, the PCR efficiencies in the experiments have a mean value of 0.9883 with standard deviation of 0.0470. In this case, the ratio change caused by different PCR efficiencies between different genes are even smaller. Therefore, relative quantification using ΔΔC_q_ method without efficiency correction could be used to determine DNA copy number.

### Copy number analysis

Quality control was performed after each PCR run. After checking amplification plots and base lines, failed reactions and outlier values (C_q_ of reference assay is greater than 32 or data point > 4 standard deviations from the mean ΔC_q_ of the four replicates) were removed. Then the C_q_ values were exported for comparative C_q_ calculation using the equations presented above. Zero was assigned to reactions with target assay’s C_q_ more than 35 and reference assay’s C_q_ less than 32. The calculation could be performed using either CopyCaller software from Applied Biosystems (Thermo Fisher Scientific) or Excel. CopyCaller provides two additional quality metrics: confidence metric and absolute z-score metric. Confidence metric estimates the confidence that the assigned copy number is the true copy number. Absolute z-score metric estimates how many standard deviations for ΔC_q_ value of one sample varies from the mean ΔC_q_ value assigned with the same copy number. The calculated copy numbers were rounded up to an integer (known as predicted copy number) for further analysis.

### Measurement of copy number discrimination

The standard deviation was used to quantify the amount of variation or dispersion in each cluster with the same assigned copy number for each test across different samples (n = 1698). As the sample size increases, the ΔΔC_q_ value tends to form approximately normal distribution. As for normal distribution, approximately 95 % of the values are within 2 standard deviations from the mean and 99.6 % within 3 standard deviations. Assuming all PCR reactions have 100 % efficiency, the ΔΔC_q_ value between one copy and two copies samples will be just 1. To be able to distinguish the difference in more than 99.6 % of the cases, the standard deviations should be less than 0.167 (allowing more than 6 standard deviations within 1). To distinguish the difference in more than 95 % of the cases, the standard deviations should be less than 0.25 (allowing more than 4 standard deviations within 1) (Additional file 1: Table S6). It is progressively more difficult to distinguish the difference between higher copy numbers since the ΔΔC_q_ value narrows.

### Assay validation

Currently there is no gold standard method available for *KIR* typing, especially with copy number information. Data generated from family-based segregation analysis and allele typing could provide information closer to the actual copy number. To validate our method, a reference panel was used including the following DNA samples which had previously been typed with standard methods (PCR-SSP/SSO):

Three extended Centre d’Etude du Polymorphisme Humain (CEPH) families’ DNA (Coriell Cell Repositories, NJ, USA) from Utah were included in this study, CEPH/UTAH Pedigree 1332, 1347 and 1416. There are 15 members in each family. Pedigree data of these families are available from dbLRC [29]. In addition, NA10832, NA10861 and NA11994 from other CEPH/UTAH families were also included because they have unusual copy numbers of *KIR* genes.

UCLA KIR Exchange panel DNA samples were from UCLA Immunogenetics Center (CA, US, http://www.hla.ucla.edu/cellDna.htm). In this study, UCLA69 to UCLA84 were selected. This cohort has consensus *KIR* gene presence and absence data generated by PCR-SSP or PCR-SSO methods from several laboratories around the world. Copy number information is not available for these samples.

Two *KIR* region fully sequenced cell lines, PGF and COX [30], as well as one sequenced CEPH cell line, NA10832 [3], were also included in this study.

A total of 1698 individuals from 339 families from the Human Biological Data Interchange (HBDI) were used to evaluate the precision of a total of 20 *KIR* quantitative PCR assays. The HBDI panel comprises Caucasian (European-ancestry) families with type 1 diabetes from the United States [31]. At present, qKAT is validated for samples of European-origin only.

### *KIR* haplotype inference in unrelated individuals

To help analyse samples, we developed a new tool, *KIR* Haplotype Identifier. *KIR* copy number data from qKAT is used as input and through matrix subtraction the program outputs the possible combinations of haplotypes for each sample. The tool is useful as a quick check for identification of novel or unconventional haplotypes in a cohort. The software processes *KIR* copy number sample files submitted by the end user. For each sample, a string of values is created through the concatenation of copy numbers provided (referred to as markersig). A second string is created comprising of a series of regular expressions for each marker (referred to as regex). Each regular expression denotes all possible haplotype values. For example, if sample 1 has markers ‘a’, ’b’ and ‘c’ each with copy numbers of 2, 1 and 1, respectively, then the markersig string would be 211 and the regex string would be (2|1|0)(1|0)(1|0), i.e. marker ‘a’ haplotype could be 2 or 1 or 0, marker ‘b’ haplotype could be 1 or 0 and so on. Each regex string is checked against *KIR* haplotype strings stored in a MySQL database. If a match is found then the associated data (e.g. haplotype, count, frequency, signature, cen motif, te1 motif) is retrieved. The haplotype pair is calculated by subtracting a matrix of the matched string from a matrix of the markersig.

## Results

### Assay validation

We tested 16 UCLA KIR exchange samples with *KIR* presence/absence data and three CEPH/UTAH families previously studied for *KIR* haplotypes. *KIR* haplotypes were determined by segregation analysis in families (see Additional file 1). For each pedigree, all non-recombinant haplotypes were identified by the Merlin program [32]. The results from *KIR* copy number assays were compared to the *KIR* data generated with previous methods [3, 24, 28]. For CEPH family samples, copy numbers were previously determined by segregation analysis of presence and absence data. Some of the duplicated genes were determined by allele typing [28] and fusion genes were determined by inter-gene PCR [3]. Apart from two exceptions described below, our results showed near complete concordance with previous data.

**Fig. 2:**
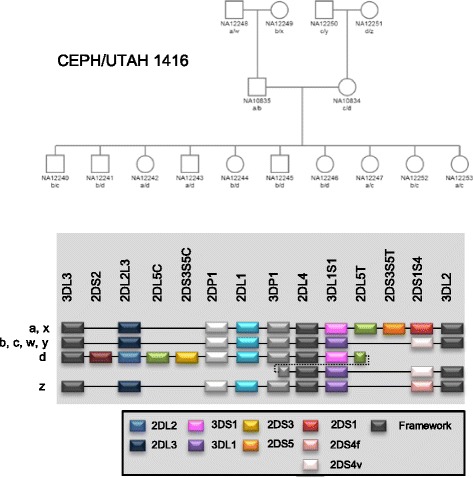
*KIR* haplotype segregation analysis in CEPH/UTAH pedigree 1416. a, b, c, d, w, x, y and z are the haplotypes deduced by segregation analysis. The gene content of each haplotype is shown. Haplotype c carries the fusion gene *KIR2DL5/3DP1* and duplication of *KIR3DP1*, *KIR2DL4* and *KIR3DL1/S1* genes [28]

In pedigree 1416, we confirmed the presence of the extended haplotype carrying duplication of *KIR3DP1*, *KIR2DL4* and *KIR3DL1/S1* genes (Fig. 2). Pedigree 1416 samples NA10834, NA12241, NA12243, NA12244, NA12245, NA12246 and NA12251 showed one less copy in *3DL1* exon 4 than in exon 9. The previous amplicon region of exon 4 was amplified and sequenced using another pair of primers (Forward 5’-CGCTGTGGTGCCTCGA-3’ and Reverse 5’ACCACGATGTCCAGGGGA-3’). Sequencing results revealed a rare allele 3DL1*056, which contains a SNP in the probe region that disrupts probe binding. The allele has only been observed in this family [33]. As factored in the original design, the second test for *KIR3DL1* does not miss this rare allele and, therefore, the allele is identified by discordant results between the two tests for *KIR3DL1*.

In pedigree 1347, sample NA11882 showed multiple copies for several genes. However only one offspring is available from this person and it seems that the haplotype carrying duplicated genes has not been transmitted to the next generation. Therefore, it was not visible by previous segregation analysis.

**Fig. 3:**
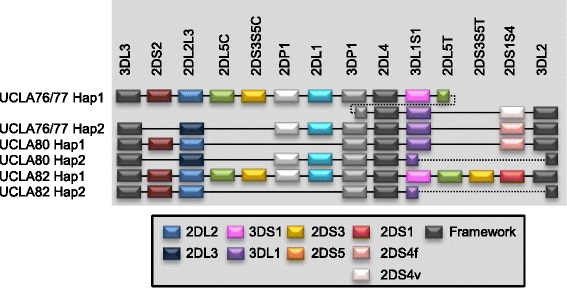
*KIR* haplotypes for UCLA KIR Exchange panel DNA samples predicted by gene copy number. UCLA76 and UCLA77 have three copies for *KIR3DP1*, *KIR2DL4* and *KIR3DL1/S1* loci and potentially carry an extended haplotype (Hap1), described previously [28]. Haplotype 2 of UCLA80 and UCLA82 have a deletion from *KIR3DL1* to *KIR3DL2*, which is similar to a haplotype carrying the fusion gene 3DL1/2v [24]

For UCLA KIR Exchange panel DNA samples, only presence and absence information was compared since the original data did not include copy number information. There was 100 % concordance between the two methods for *KIR* presence and absence data. However, additional information was given by copy number data. For example, UCLA76 and UCLA77 have three copies for *3DP1*, *2DL4* and *3DL1/S1* loci. Potentially these two samples carry an extended haplotype, described previously [28] (Fig. 3). UCLA80 and UCLA82 have a deletion from *3DL1* to *3DL2*, which is similar to a haplotype carrying the fusion gene *3DL1/2v* [24] (Fig. 3). For NA10861, NA11994 and the sequenced cell line PGF, COX and NA10832, the copy numbers calculated from our assay were the same as predicted from previous analysis [3, 30].

CNV is seen for *LILRA3* and *LILRA6* loci but not for other *LILR* genes [34]. Copy number of *LILRA6* had previously been determined for the CEPH family samples using Taqman CNV assays [35]. Our results showed complete concordance with this previous data (Additional file 1: Table S7).

### Measurement of copy number clustering

**Fig. 4:**
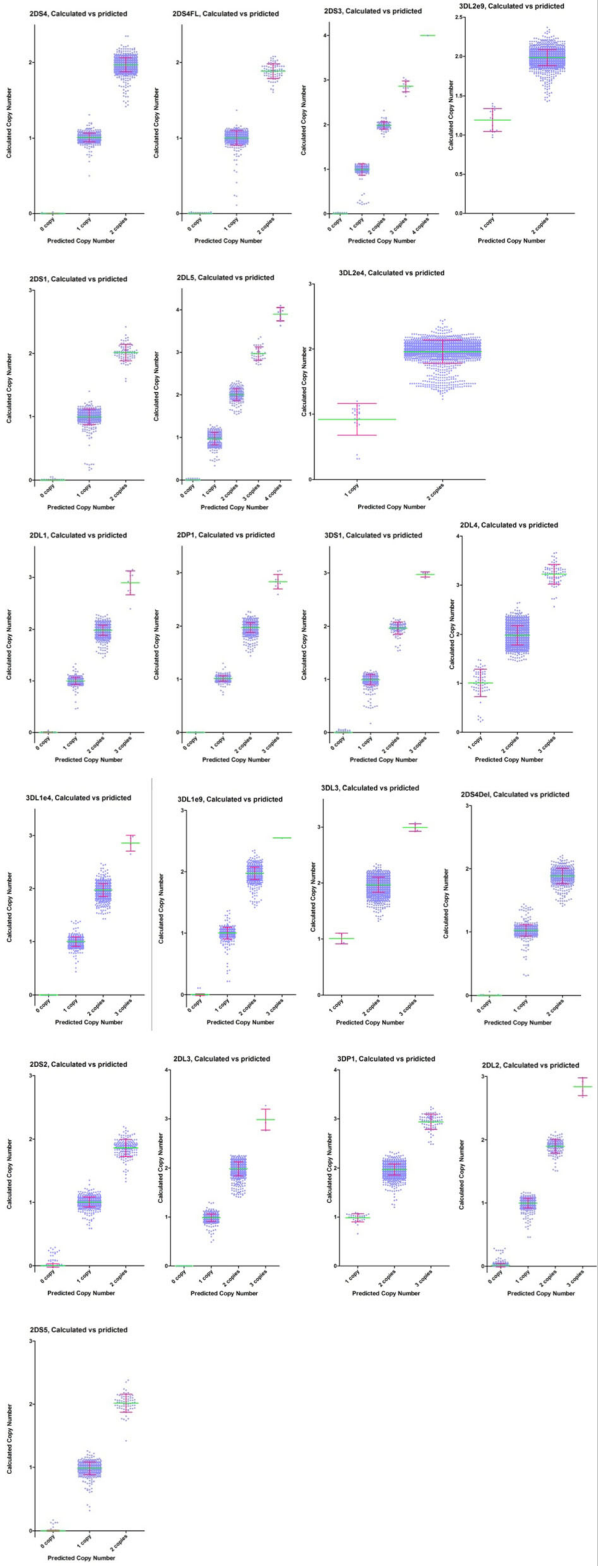
Calculated copy number plotted against predicted copy number. Each cluster represents samples assigned with the same copy number. The line and error bar represent mean value and standard deviation of each cluster. The *KIR* gene copy numbers can be determined empirically or, as in this example, by using algorithms incorporated into the CopyCaller software

Representative examples of clustering results derived for all the *KIR* are given in Fig. 4. The standard deviation in each cluster with the same assigned copy number was used to evaluate ability of clear copy number discrimination in the 20 *KIR* quantitative PCR assays. A total of 1698 DNA samples were used in this analysis (Additional file 1: Table S8). It was possible to confidently distinguish between 3 and 4 copies or even up to 5 copies with the data generated from *KIR* assays. Moreover, most of the data sets displayed a tighter distribution than a Gaussian distribution (D’Agostino–Pearson normality test). In this situation, the standard deviation usually overestimates the data variability.

### KIR haplotype inference in unrelated individuals

Family-based segregation analysis can be used to determine gene content on haplotypes. However, in unrelated samples assigning a gene to a haplotype is indefinite when copy number is greater than one for any given locus. For example, an individual carrying two copies of *KIR3DS1* and one copy of *KIR3DL1* could have two copies of *KIR3DS1* on one haplotype and one copy of *KIR3DS1* on the other haplotype. Alternatively, the individual could have one copy of *KIR3DS1* and *KIR3DL1* on the same haplotype with the second copy of *KIR3DS1* on the other haplotype. Approaches for inferring *KIR* haplotype from copy number are detailed in Supplementary Material (Additional file 1: Tables S9–S11). To facilitate analysis of samples for *KIR*, an online tool was developed using Perl and MySQL called ‘KIR Haplotype Identifier’ (http://www.bioinformatics.cimr.cam.ac.uk/haplotypes/) (Additional file 1: Figure S5). This is a tool for imputing haplotype pairs using observed copy number for each *KIR* loci to help resolve *KIR* haplotypes from unphased genotypes of unrelated individuals. The default haplotype library used by this program is based on European-origin *KIR* gene data but analysis can be carried out using your own *KIR* haplotype frequency data. This is important when analysing a different population, otherwise the software may not give a proper representation of the samples. The tool outputs the possible combinations of haplotypes for each sample based on the gene content of all haplotypes supplied in the haplotype file. Three output files are generated. The first file (Haplotype Results) lists all possible haplotype pairs for each sample, each haplotypes frequency (from the haplotype file) and the predicted combined frequency of each haplotype pair (Additional file 1: Figure S5). The second file is in the same format as Haplotype Results; however, it lists only haplotypes with the highest combined frequency (Haplotype 1 Frequency × Haplotype 2 Frequency) for each sample. The third file (Log file) contains a list of samples where haplotype pairs could not be assigned. In these cases, possible single haplotypes are listed per sample. These results can be visualised by a ‘KIR Haplotype Resolution Drawing Tool’ developed using R (Additional file 1: Figure S6). The script is available upon request.

## Discussion

In this paper, we describe a *KIR* typing method based on real-time PCR. This method is able to detect the total number of copies of each *KIR* locus. Clear discrimination between 0, 1, 2, 3 or even 4 copies could be obtained using this method. We extended the approach to *LILR* loci, demonstrating that the qKAT approach can be used to analyse other mCNV loci.

This method is high-throughput and cost-effective. Using a Roche LightCycler 480 real-time PCR instrument with a 384-well Thermal Block Cycler we could complete our PCR assay in 65 min. A Twister II Plate Handler was used as an automation robotics system (Additional file 1: Figure S7). A MéCour Thermal Plate Stacker was used to keep the stacked plates constantly at 4 °C. With automation, around 22 plates comprising 8448 reactions can be finished within 24 h. Since our full *KIR* typing assay for each sample requires 40 reactions in total (including quadruplicates), this system can produce full *KIR* typing for around 210 samples every day.

Copy number information provided by quantitative PCR may be essential for accurate *KIR* determination. Accurate genotype data are required for population genetic studies and gene dosage effects [36]. Unlike standard genotypes, some *KIR* genes (e.g. *KIR2DL5*, *KIR2DS3* and *KIR2DS5*) can be missing and actual genotype cannot be resolved without copy number information (e.g. –A and AA). Furthermore, recently discovered structural variations make typing even more difficult [3, 24, 28]. For truncated haplotypes carrying a multi-locus deletion, conventional methods can only detect them in a homozygote. For extended haplotypes carrying duplicated loci, typing at the allelic level may be helpful when the multiple alleles are different to each other. Specially designed inter-gene PCRs are useful approaches [3, 24, 37] but from our data it seems there may be many more truncated and extended haplotypes [38]. Nevertheless, none of the approaches could provide precise genotype without family data. Recently, pyrosequencing has been used for *KIR* typing and this can also provide copy number information although there are throughput and cost limitations [24, 39].

Accuracy is extremely important for quantification. We have shown that it is possible for real-time PCR to accurately determine the copy number from genomic DNA. Reference DNA samples were used to validate the accuracy and a large panel of families (1698 samples) to evaluate the precision. Since most CNVs follow Mendelian inheritance, family information can be used to infer copy number in each homologous chromosome after the total copy numbers are obtained from quantitative PCR. This method has been shown to enhance the accuracy of CNV detection [40]. For example, in CEPH family 1347, copy number information assisted in the deduction of gene content for all haplotypes when family data were insufficient to resolve haplotypes for all members with *KIR* presence/absence data. Our method could be further improved by using probabilistic models to increase confidence of chromosome-specific copy number estimates using family information [41]. This approach can be used for the future development of linkage and association tests that require chromosome-specific copy number information. However, like any other PCR-based method, highly polymorphic sequences always pose challenges for designing primers and probes. As we found with the 3DL1*056 allele in family CEPH1416, there is always the possibility that some rare alleles may be missed due to polymorphism. The primer and probes were designed to avoid known polymorphism in their annealing sites (Additional file 1: Tables S3 and S5) but as more alleles are described, care should be taken to continually review the assays and redesign the primers/probes as required. If an assay is disrupted by a rare SNP (true allele dropout) this will be identified by the loss of linkage with an adjacent gene that is known to be in high linkage disequilibrium; all *KIR* loci have another *KIR* locus in tight linkage or have an expected copy number, e.g. framework genes are usually always two copies. One can, therefore, check the data against these predefined ‘standard *KIR* haplotype rules’ (Additional file 1: Table S13) to identify unexpected results and these samples can be further investigated. Alternatively, inconsistencies can be found using the KIR Haplotype Identifier online tool through the appearance of an unusual haplotype in the results. In rare instances when confirmation is required, a second set of assays for each gene can be used for verification (Additional file 1: Table S14).

There are opportunities for further development of the copy number assay. For example, triplex real-time PCR was used in this assay, but it may be possible to achieve up to heptaplex real-time PCR [42] to improve the throughput. Inclusion of an additional reference gene or multicopy reference could provide superior normalisation for DNA input and avoid potential effects of local genomic changes to the reference gene. Supporting our current choice of reference gene, in our screening with qKAT we have not yet identified a sample exhibiting altered *KIR* copy number across all *KIR* loci, including framework genes, indicative of a genomic alteration to the reference gene. Currently, there are other methods to discriminate gene copy number, e.g. DNA microarray, multiplex ligation-dependent probe amplification (MPLA), branched DNA testing, paralogue ratio test (PRT), digital PCR [43] and next generation sequencing (NGS). In addition, *KIR* haplotyping can be achieved through dye-terminator sequencing of *KIR* gene amplicons [44]. Comparing current throughput, cost and complexity of assay setup, quantitative PCR has advantages over the others for *KIR* copy number analysis. Highly repetitive genomic intervals with long stretches of identical sequence, as in the *KIR* locus, have been less amenable to NGS. The present short read lengths obtained by NGS, or the current inaccuracy of long-read length single molecule sequencing, makes sequence assembly and phasing (haplotype-resolution) problematic for characterisation of mCNV loci, especially when more than two copies of a gene are present. As NGS methods improve and become cheaper, we anticipate that this approach will be useful, particularly at increased scale and for precise typing of *KIR* alleles at the nucleotide level. The two approaches will complement each other and be useful for cross-validation [45]. qKAT offers a simple solution for, as example, initial assessment *KIR* disease association at the gene-level or haplotype-level before investing more time in complex analysis at the allele-level. Once an association has been established, allele resolution typing could be informative if a sufficient number of samples are available for statistically powered analysis. qKAT is simple, one-step and flexible, in that a single gene, or combinations of genes, can be typed alone at minimal cost or as required (e.g. *KIR* A/B haplotype-defining genes). To date, 21 published studies (comprising >20,000 samples in total) including investigations of *KIR* disease association, function, expression and imputation have utilised the method [13, 31, 34, 38, 43, 45–60]. A *KIR* typing service using qKAT has also been established at the Addenbrooke’s Hospital Histocompatibility and Immunogenetics (Tissue Typing) laboratory in Cambridge (UK).

## Conclusion

This simple, high-throughput and cost-effective direct *KIR* typing method can be used for disease association studies. In these studies, large numbers of cases and controls are usually needed for *KIR*-*HLA* interaction analysis. Therefore, this method allows analysis of large-scale studies that were previously labour-intensive, time-consuming and cost-prohibitive. The underlying strategy of qKAT offers a model for analysing any other highly diverse genomic regions of interest.

## Additional file

Additional file 1: Supplementary figures S1–S9, supplementary tables S1–S14. (DOCX 2712 kb)

## Abbreviations

BHQ: Black hole quencher
CEPH: Centre d’Etude du Polymorphisme Humain
HBDI: Human Biological Data Interchange
HLA: Human leukocyte antigen
HWE: Hardy–Weinberg Equilibrium
KIR: Killer immunoglobulin-like receptor
LRC: Leukocyte receptor complex
MALDI-TOF: Matrix-assisted laser desorption/ionisation time-of-flight mass spectrometry
mCNV: Multi-allelic copy number variation
MPLA: Multiplex ligation-dependent probe amplification
NAHR: Non-allelic homologous recombination
PCR-SSO: Sequence-specific oligonucleotide PCR
PCR-SSP: Sequence-specific primed PCR
qKAT: Quantitative KIR semi-automated typing
