# Supplementary material for: qKAT: a high-throughput qPCR method for KIR gene copy number and haplotype determination
Source: Genome Med. 2016 Sep 29;8:99. doi: 10.1186/s13073-016-0358-0 (PMC5041586; doi:10.1186/s13073-016-0358-0)
Supplement: Additional file 1: — Supplementary figures S1–S9, supplementary tables S1–S14. (DOCX 2712 kb) [file 13073_2016_358_MOESM1_ESM.docx]

# **Additional file 1**

**qKAT: A high-throughput qPCR method for KIR gene copy number and haplotype determination**

Jiang W, Johnson C, Simecek N, López-Álvarez MR, Di D, Trowsdale J, Traherne JA


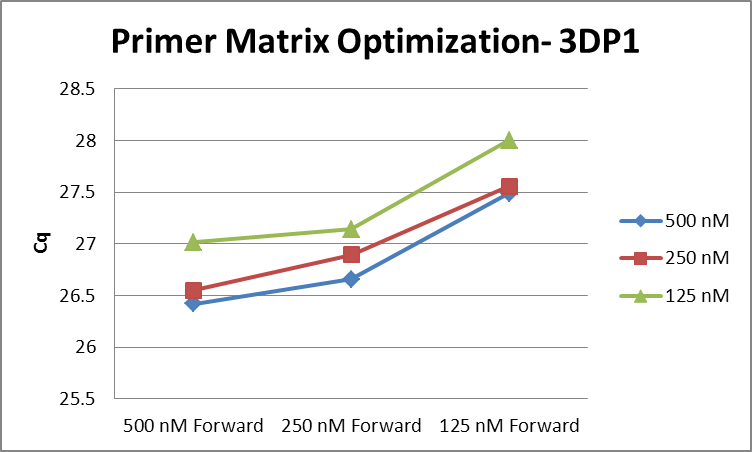


Forward: 250nM, reverse: 250 nM.


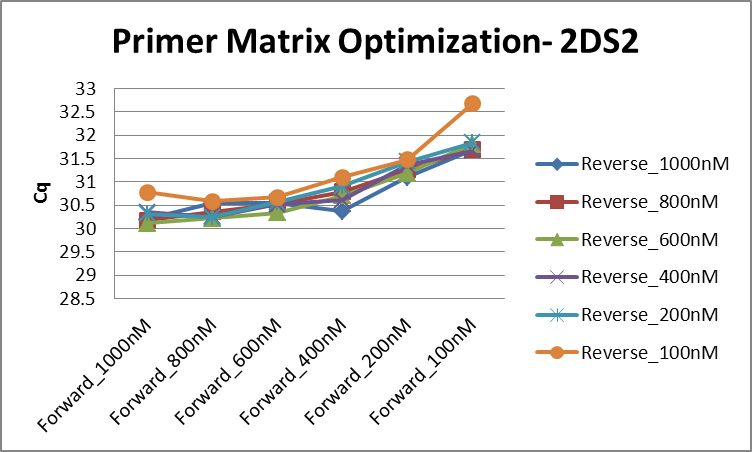


Forward: 400nM, reverse: 400 nM.


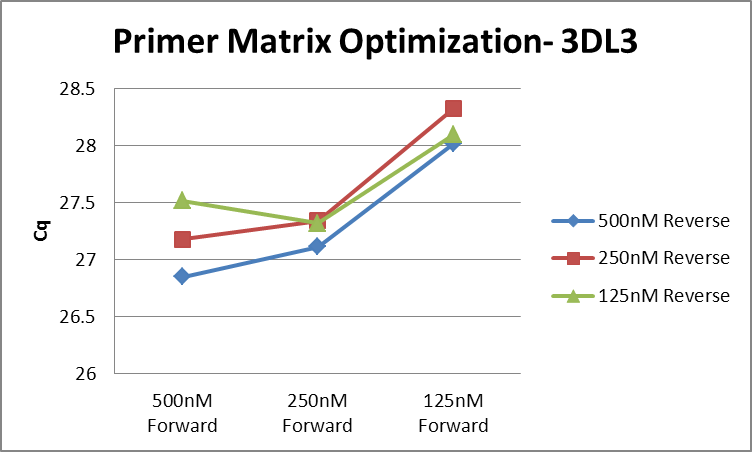


Forward: 500nM, reverse: 500 nM.


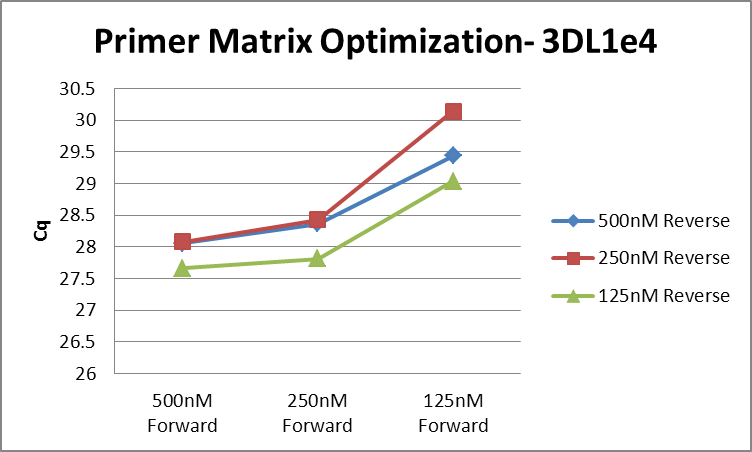


Forward: 250nM, reverse: 125 nM.


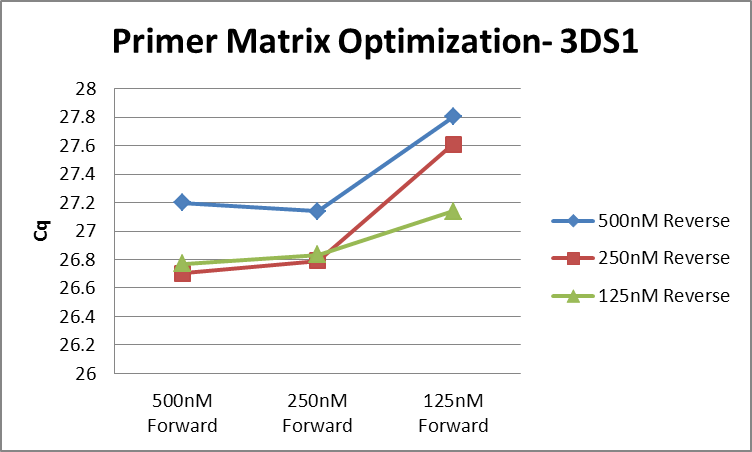


Forward 250 nM, reverse 250 nM.


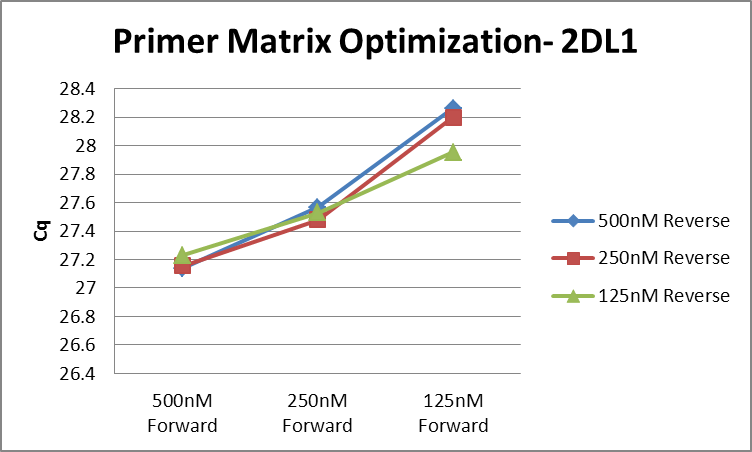


Forward 500nM, reverse 125nM.


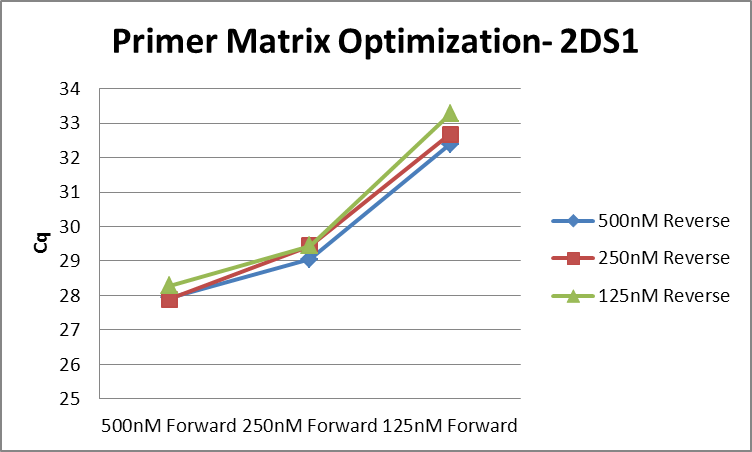


Forward: 500 nM, reverse 250 nM.


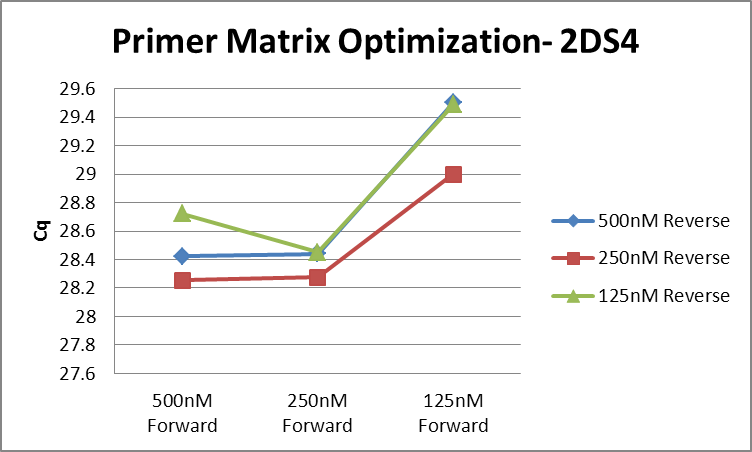


Forward: 250 nM, reverse: 250 nM.


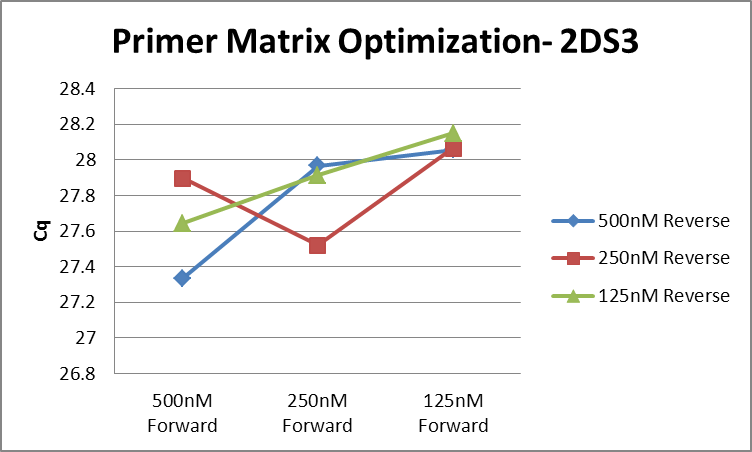
Forward: 250 nM, reverse 250 nM.


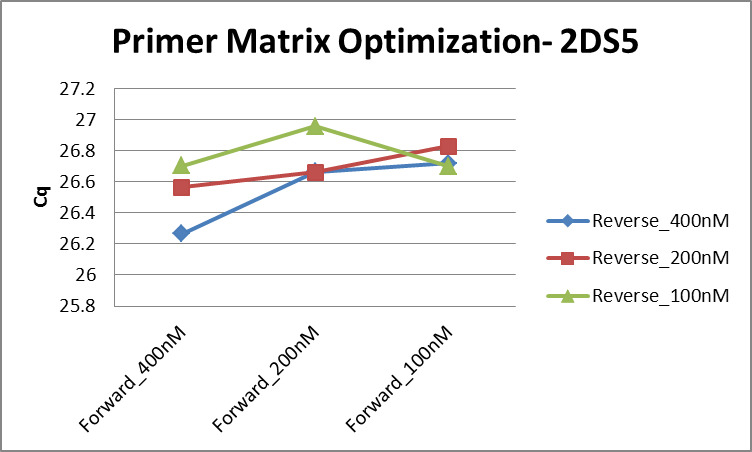


Forward: 200 nM, reverse: 200 nM.


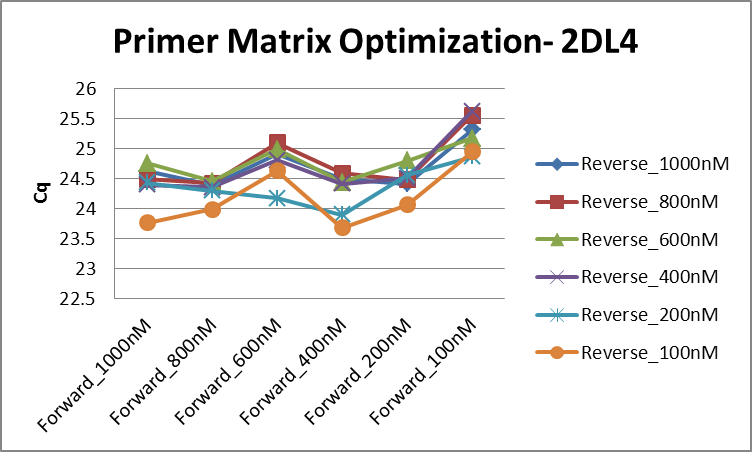


Forward: 200 nM, reverse: 200 nM.


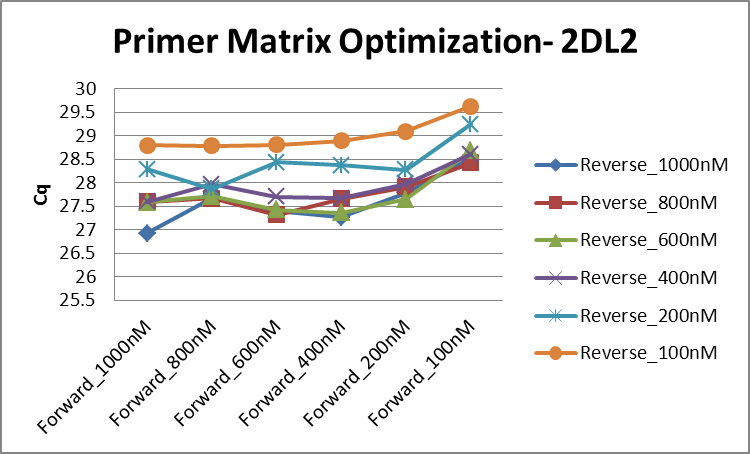


Forward: 400 nM, reverse: 600 nM.


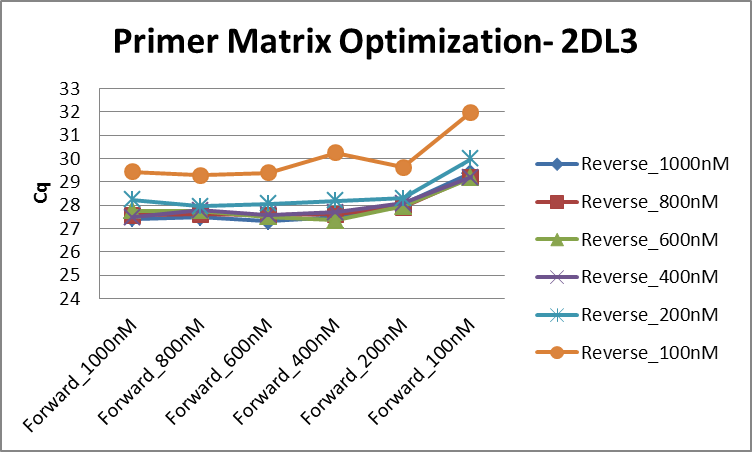


Forward: 400nM, reverse 400 nM.


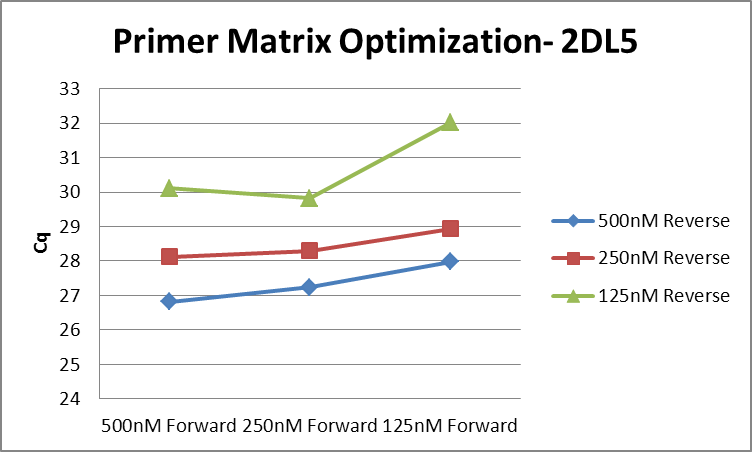


Forward: 500nM, reverse 500 nM.


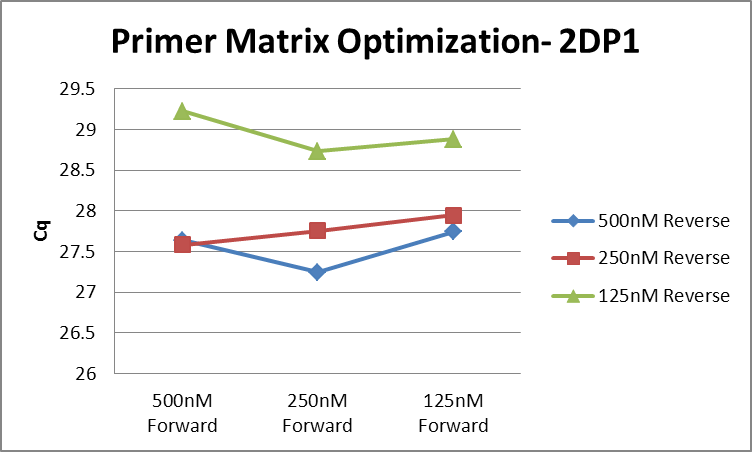


Forward: 250nM, reverse 500 nM.


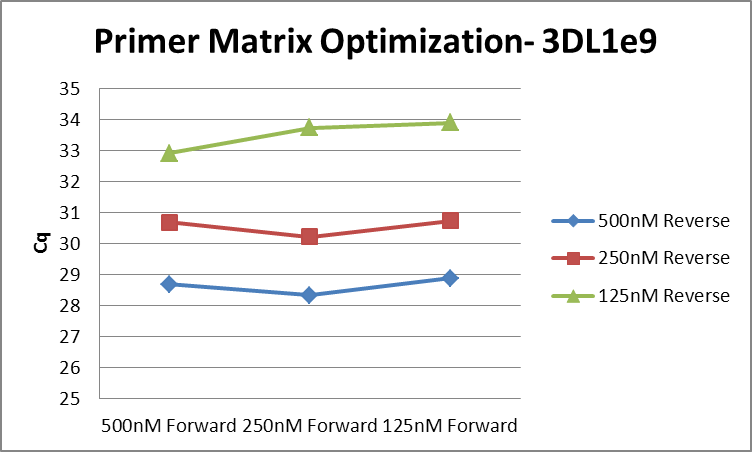


Forward: 250nM, reverse 500 nM.


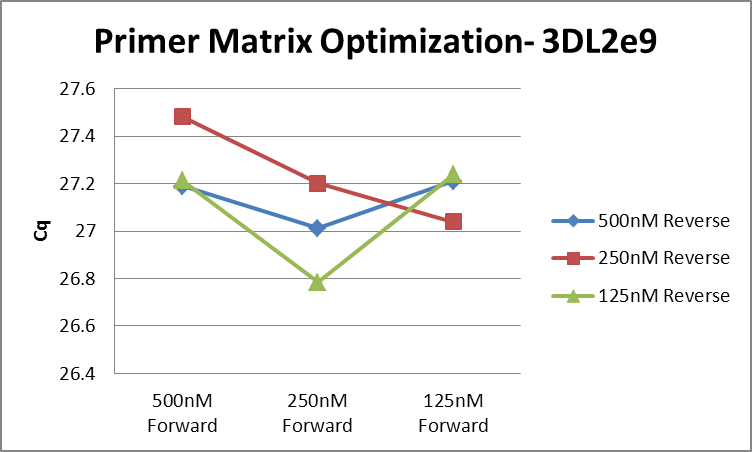


Forward: 250nM, reverse 125 nM.


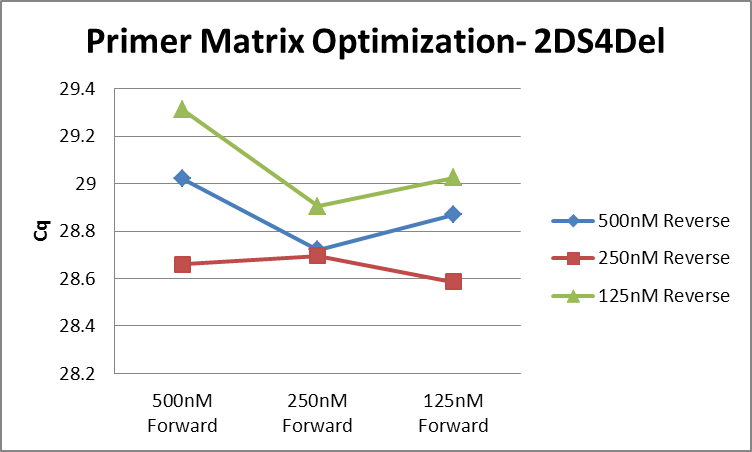


Forward: 250nM, reverse 250 nM.


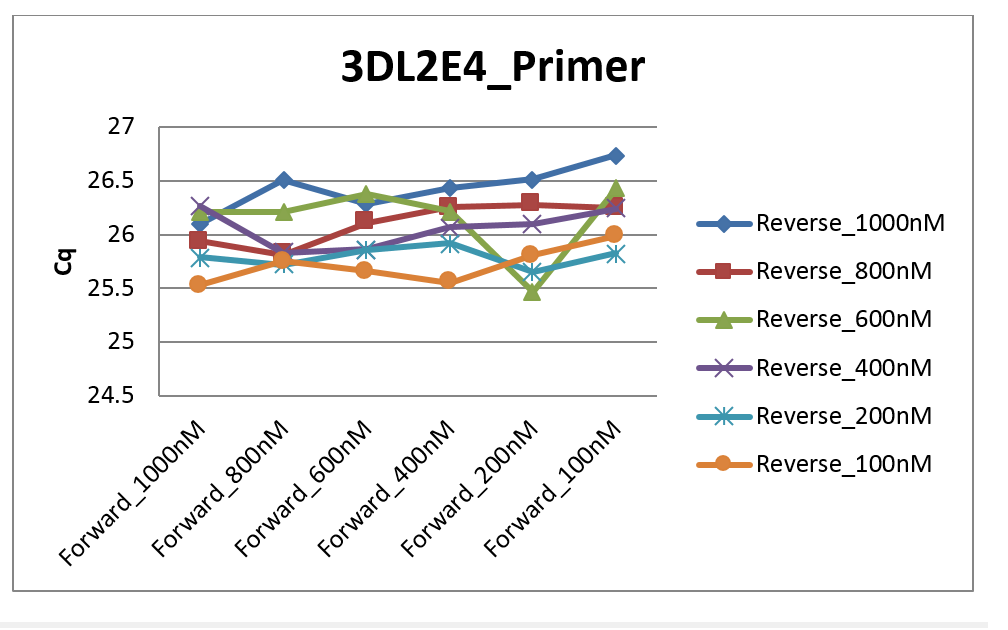


Forward: 200nM, reverse 200 nM.
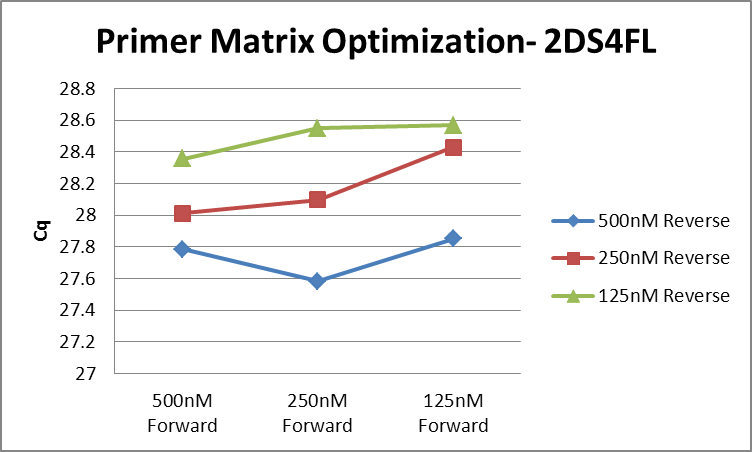


Forward: 250nM, reverse 500 nM.


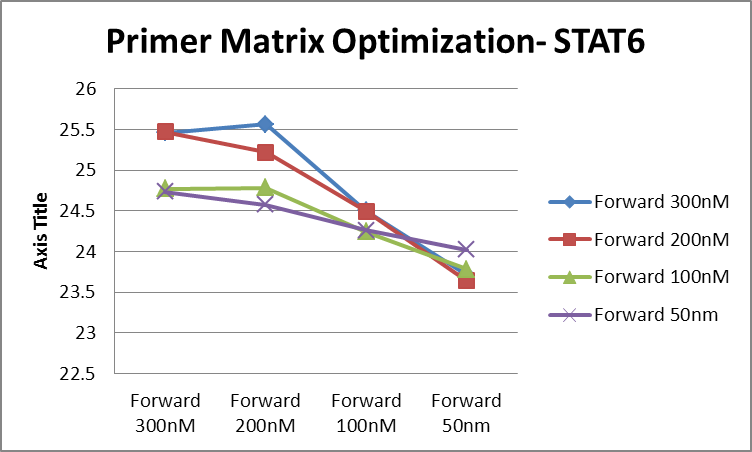


Forward: 200nM, reverse 200 nM.

**Supplementary Figure 1: Primer concentration optimization using SYBR Green.**

The determined optimal primer pair concentrations were checked for high performance in multiplex assays. The primer concentrations optimizations were carried out using different combinations of forward and reverse primers in a matrix format. The x-axis = different forward primer concentrations, and y-axis = C_q_ value of each reaction. As given in the legend, different plots represent different reverse primer concentrations. Primer concentration of 125 nM, 250 nM and 500 nM were tested (or otherwise stated in the figure) using previously verified positive samples. The optimal concentration of each primer varies. The primer pair with highest PCR performance (the lowest C_q_ value) and the lowest possible concentrations were selected. Melting curves were also checked to confirm that there was only a single peak for each amplification to ensure there were no primer dimers or non-specific amplification. Five nanograms of genomic DNA from donors positive for the gene being tested (previously verified by PCR-SSP) were used.

The specificity and sensitivity was increased by controlling the primer and probe concentrations since reducing the total oligonucleotide in the assay prevents the individual oligonucleotides interfering with each other. Using SYBR Green I was a convenient and inexpensive approach to examine the functionality of the primers and to provide the quantification measures for the PCR reaction.

Probe P4a concentration optimization.

Series 1 using 3DP1 primers; series 2 using 3DL3 primers.

Probe P4b concentration optimization.

Series 1 using 3DS1 primers; series 2 using 2DS5 primers.

Probe P5b concentration optimization.

Series 1 using 2DL4 primers; series 2 using 2DS4 primers.

Probe P9 concentration optimization.

Series 1 using 2DL3 primers; series 2 using 3DL2e9 primers.

Probe P4a concentration optimization.

Series 1and 2 using different samples.

# **Supplementary Figure 2: Probe concentration optimization**

The probe concentration for each assay was optimized after the optimal primer concentration was determined from the primer titration assays. Probe concentrations from 50 nM to 500 nM were tested for *KIR* assays. The *KIR* probes were tested using the same positive DNA sample with different primers that amplify the same exon. The reference gene (*STAT6*) was verified using different DNA samples but the same primer. The lowest possible probe concentrations that produced an acceptable C_q_ value (~26-28) were selected.


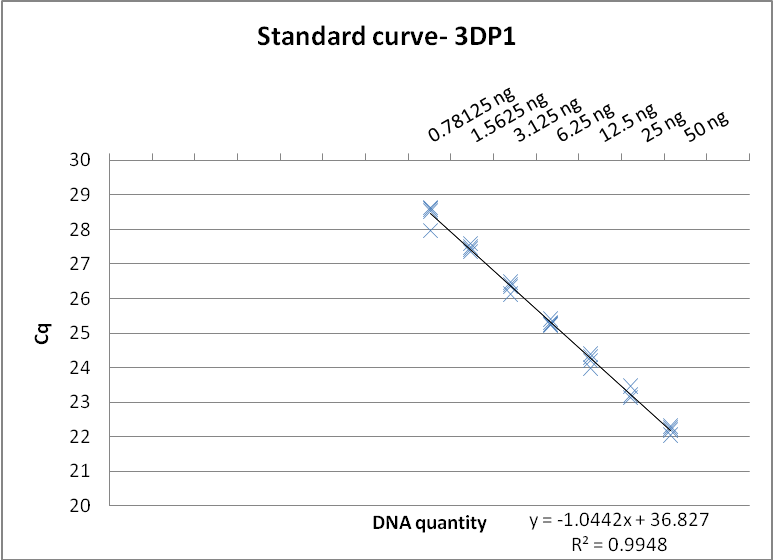


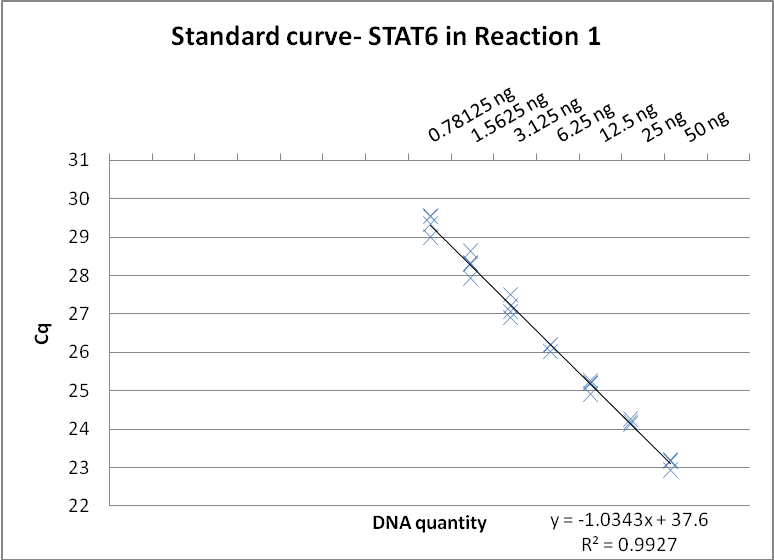


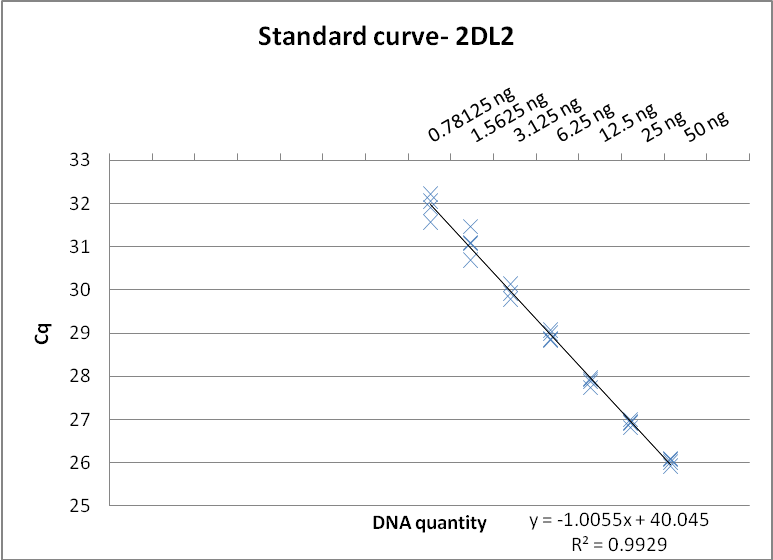


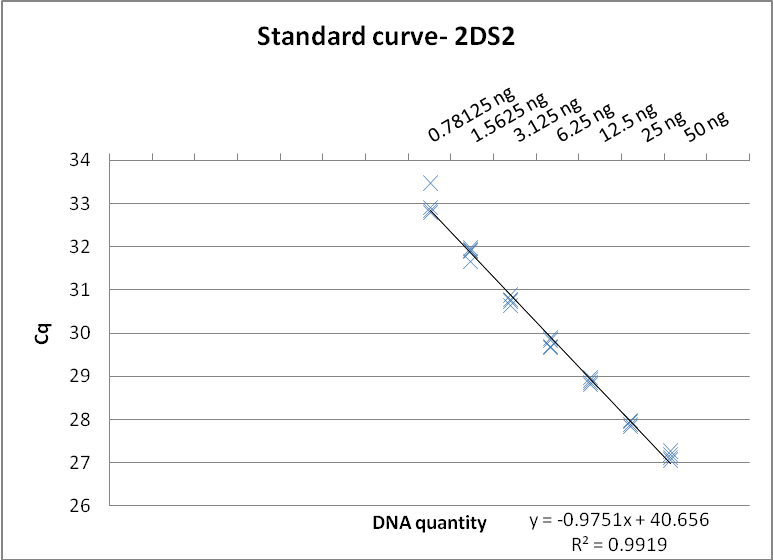


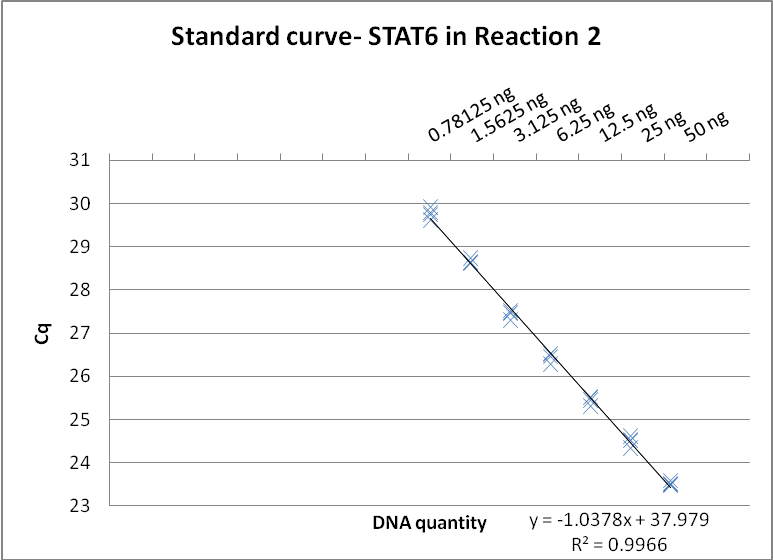


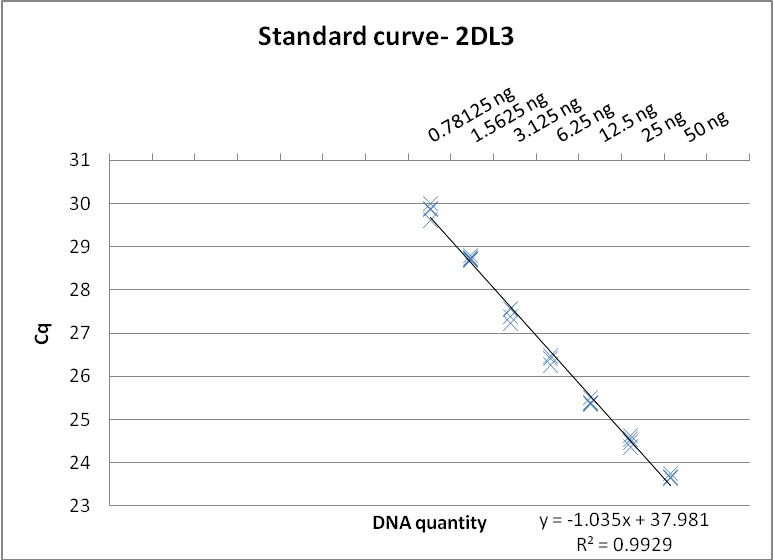


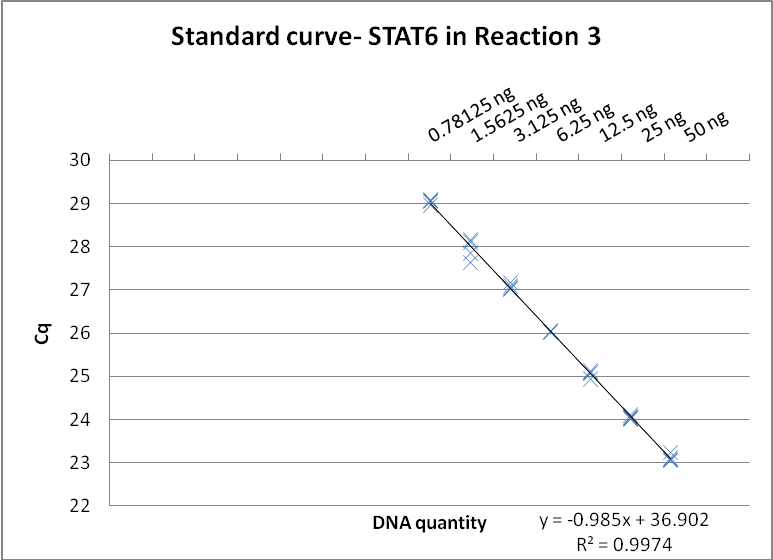


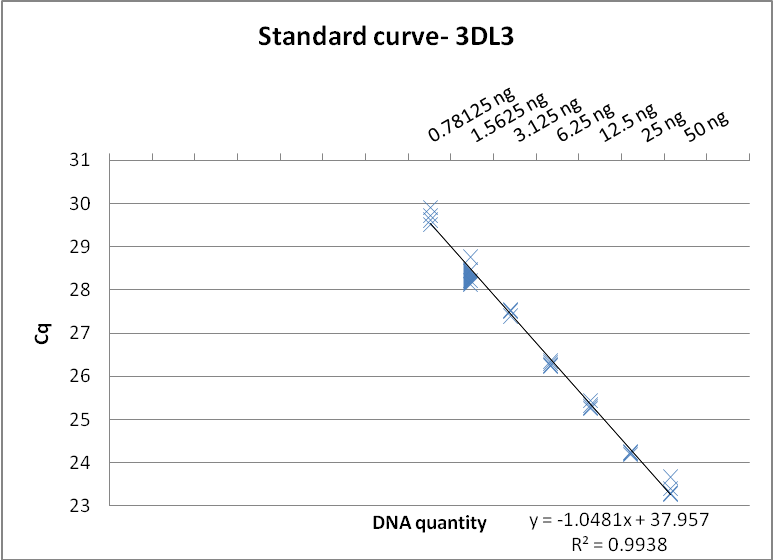


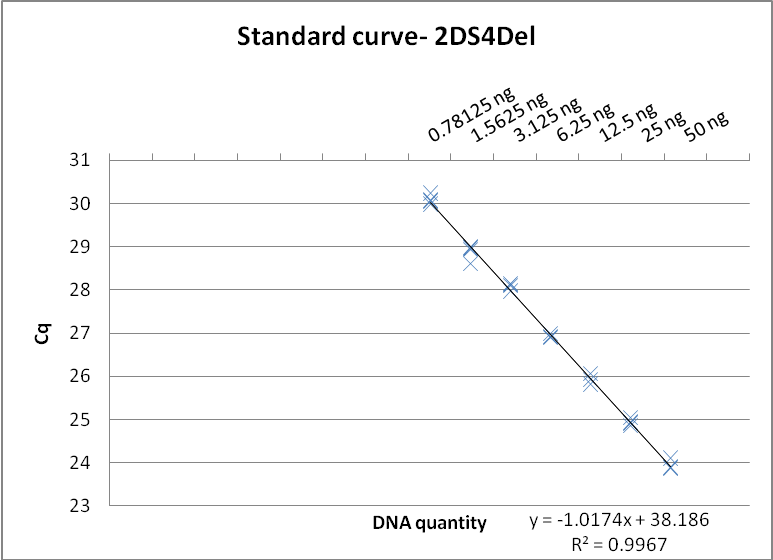


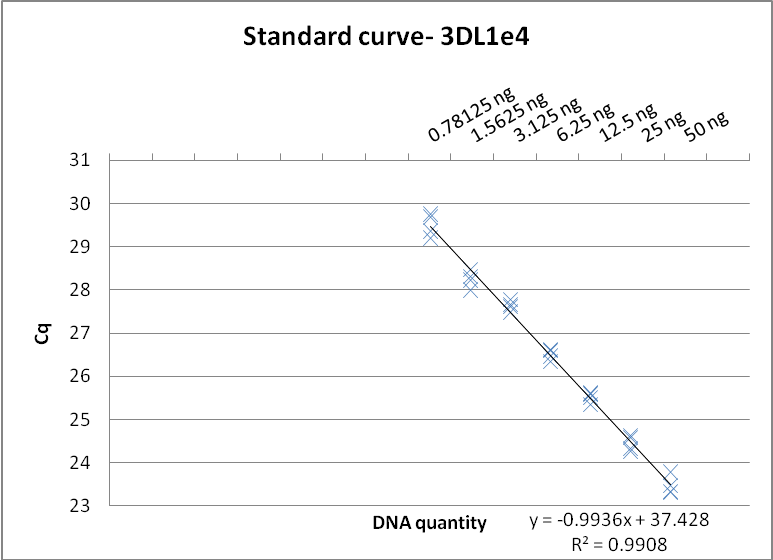


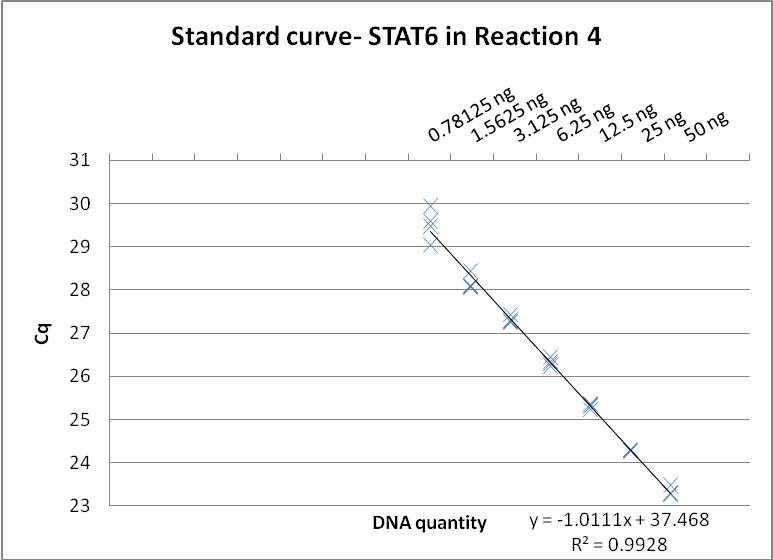


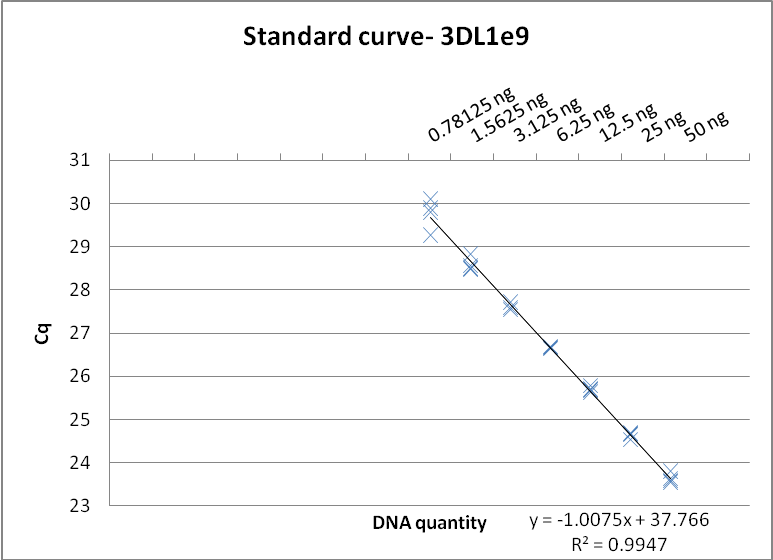


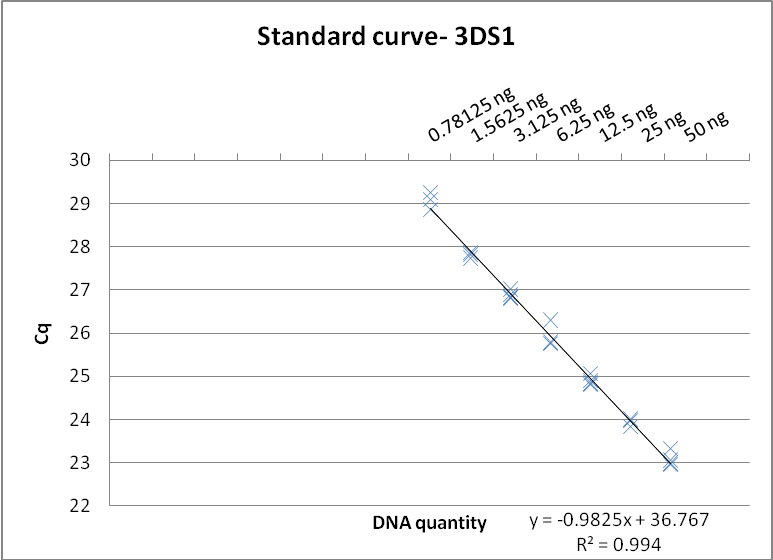


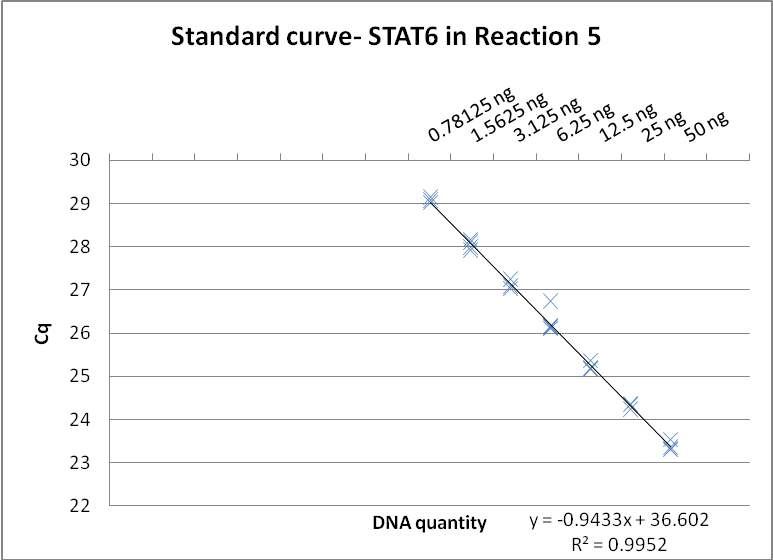


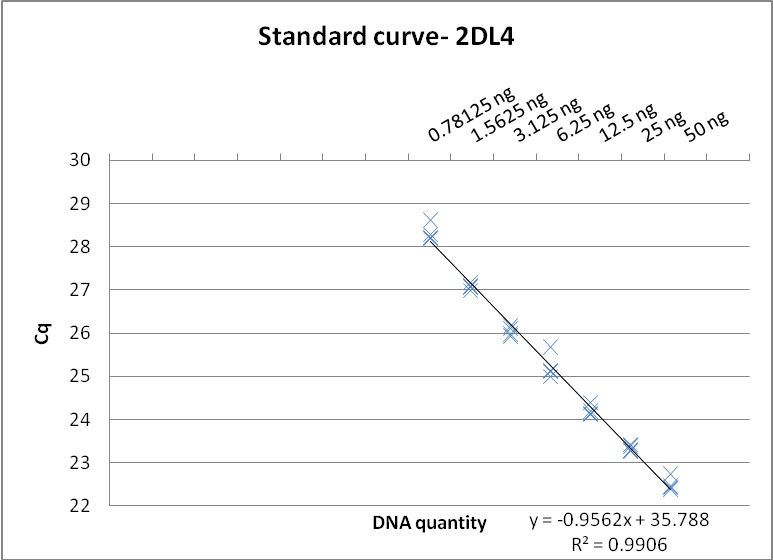


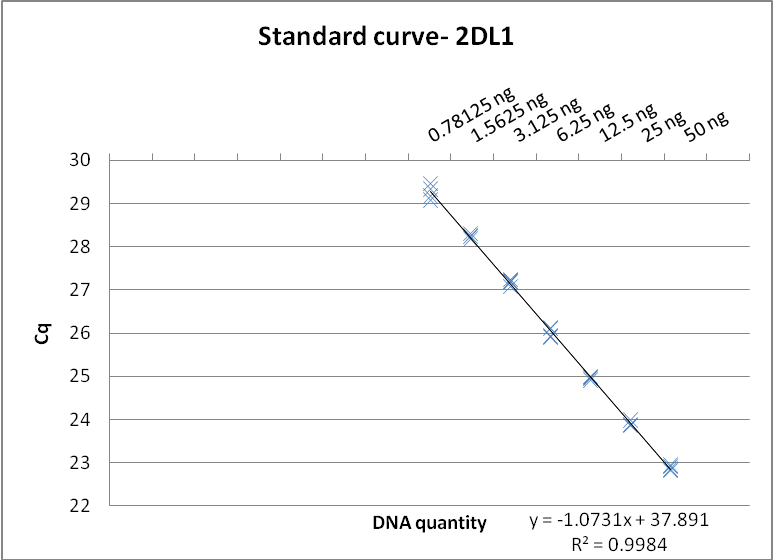


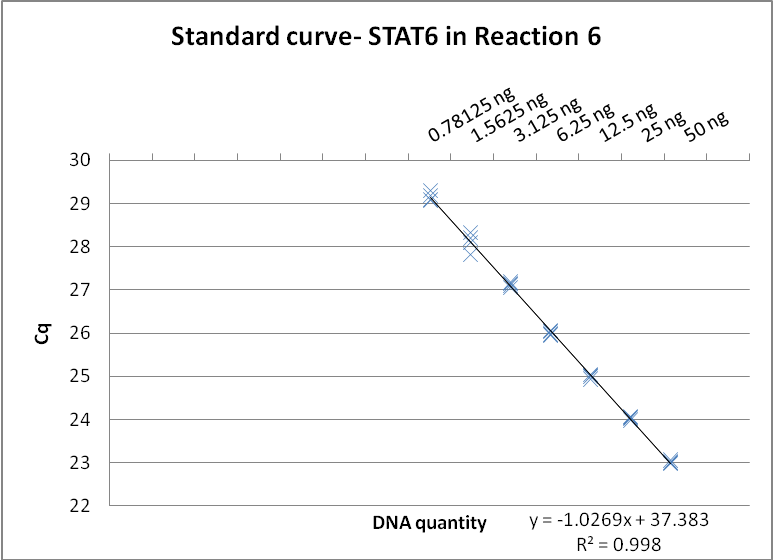


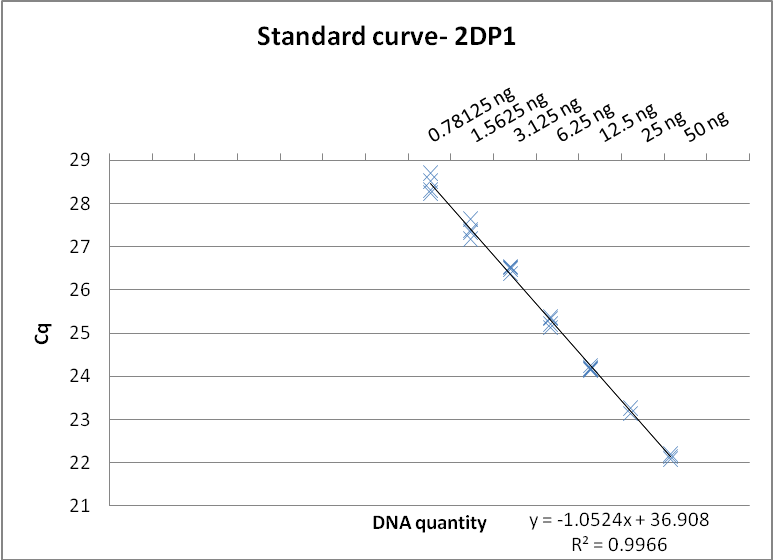


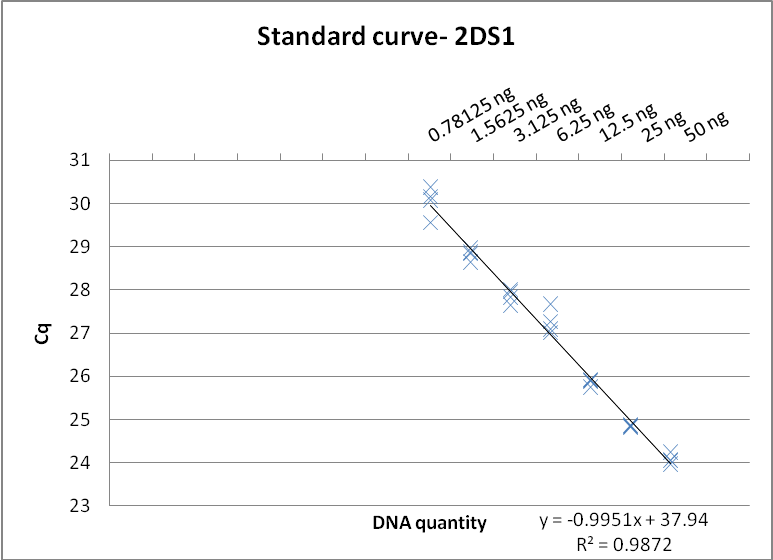


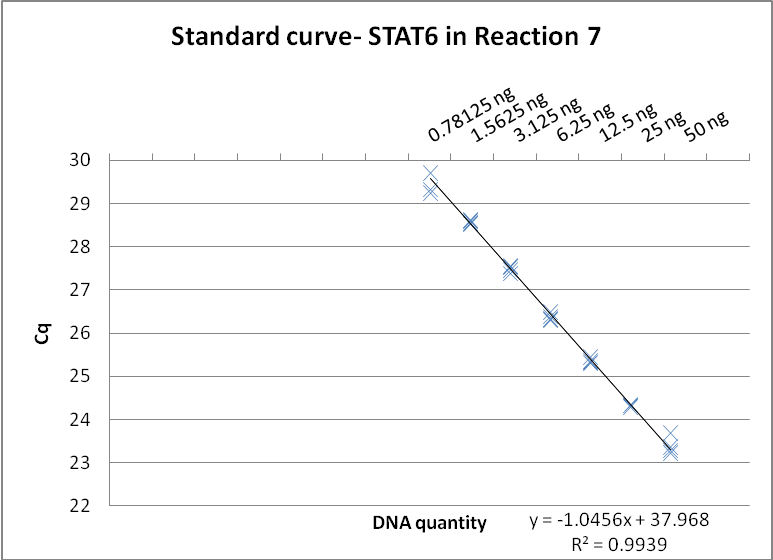


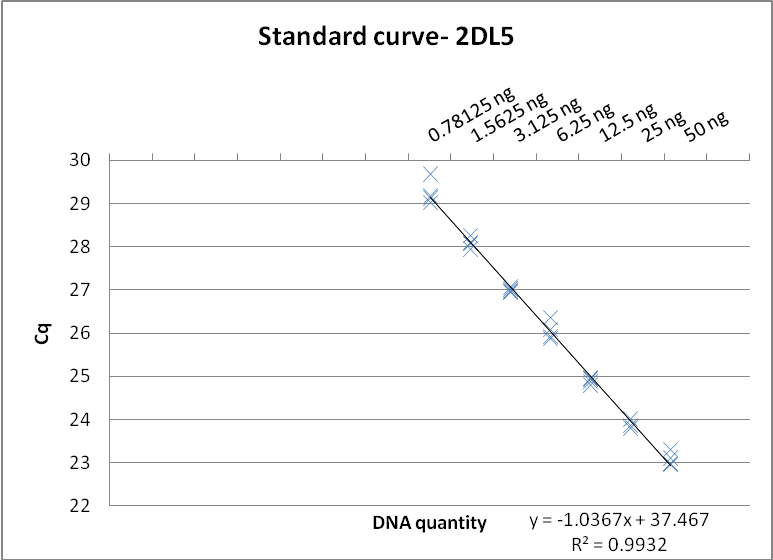


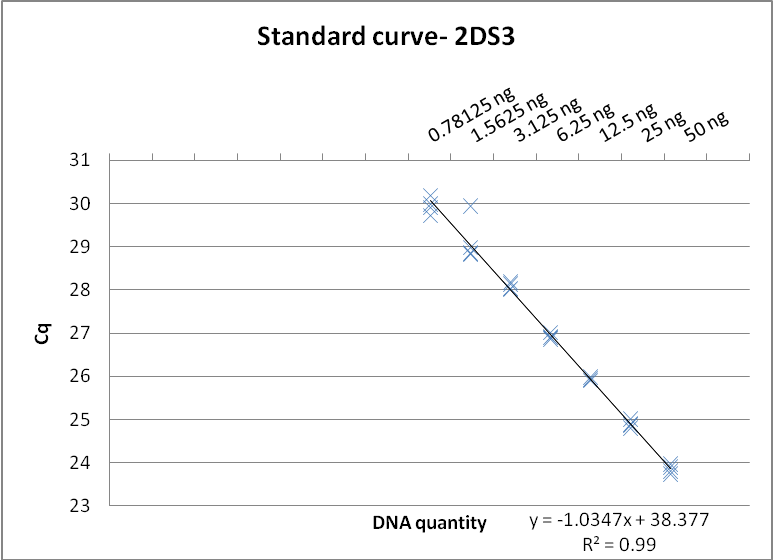


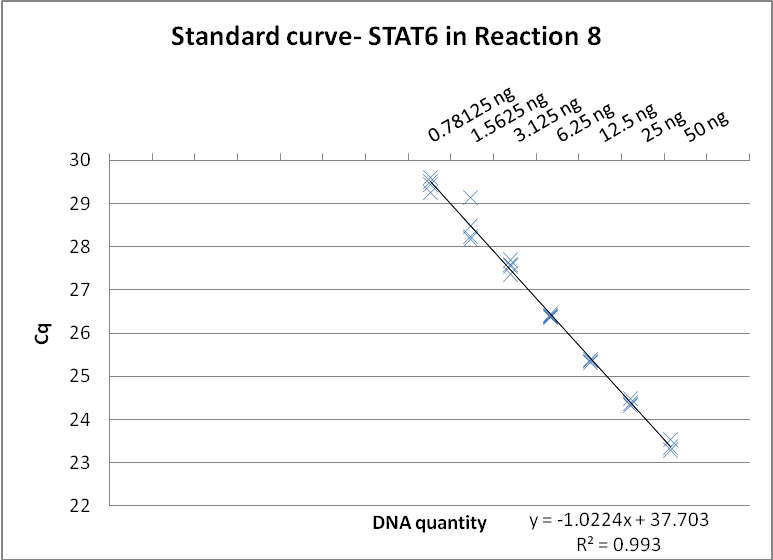


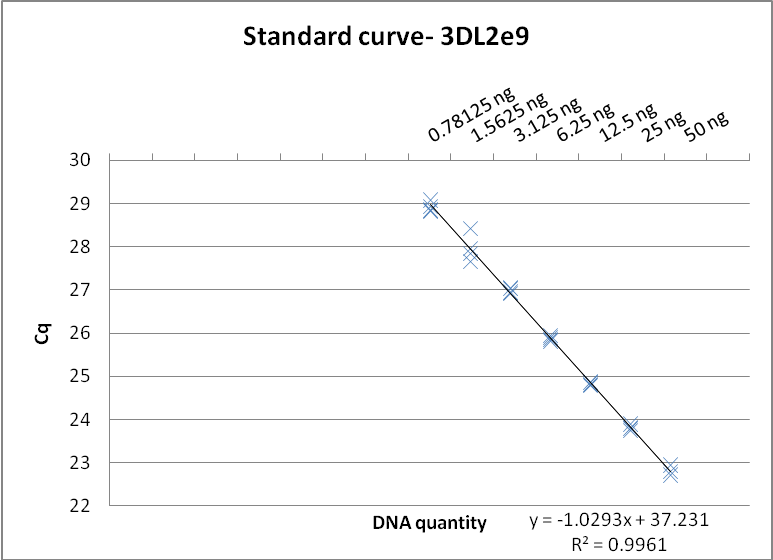


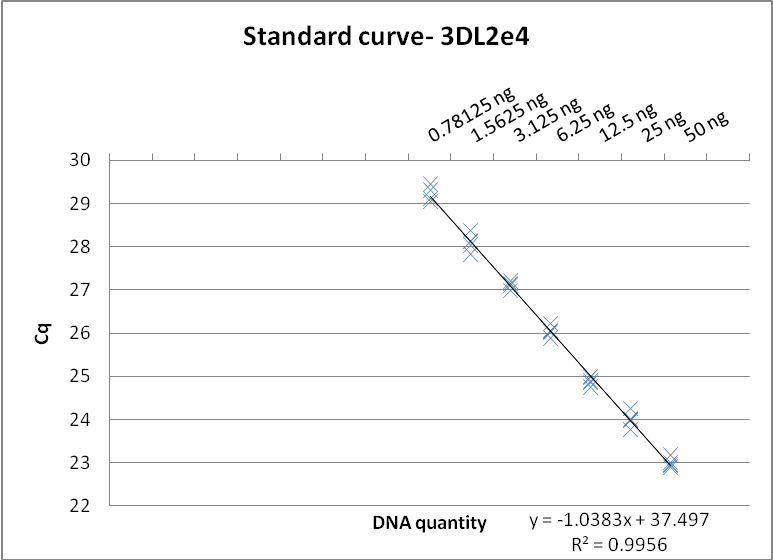


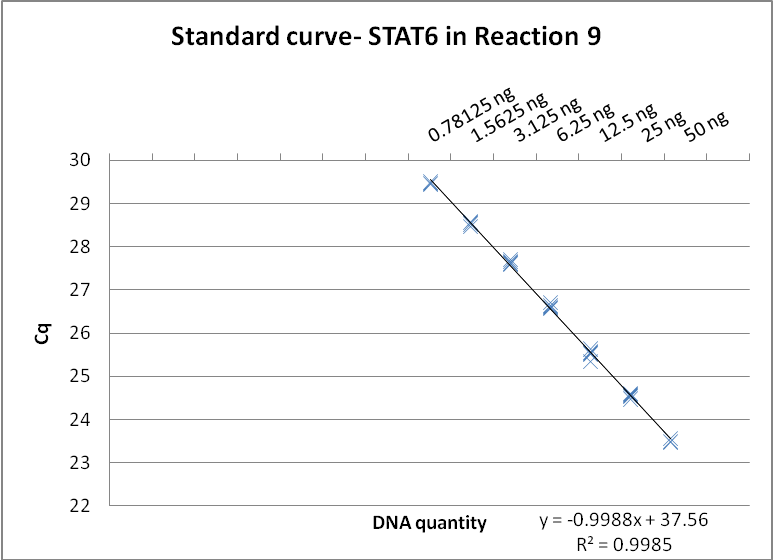


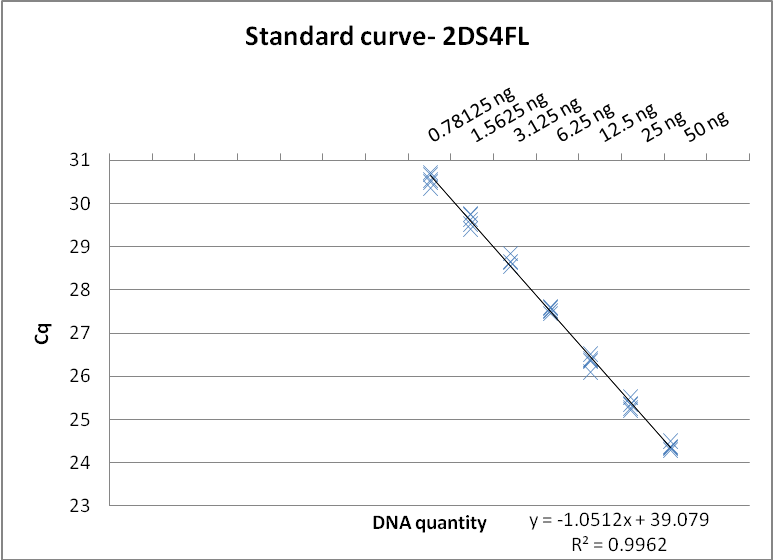


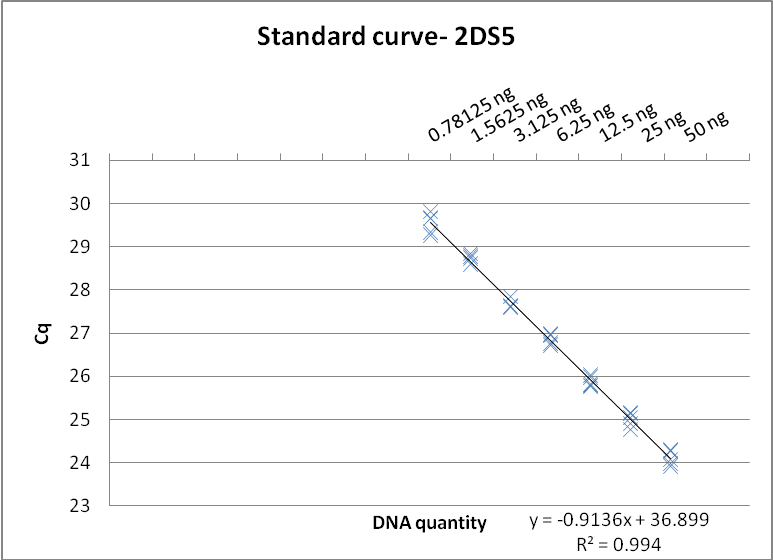


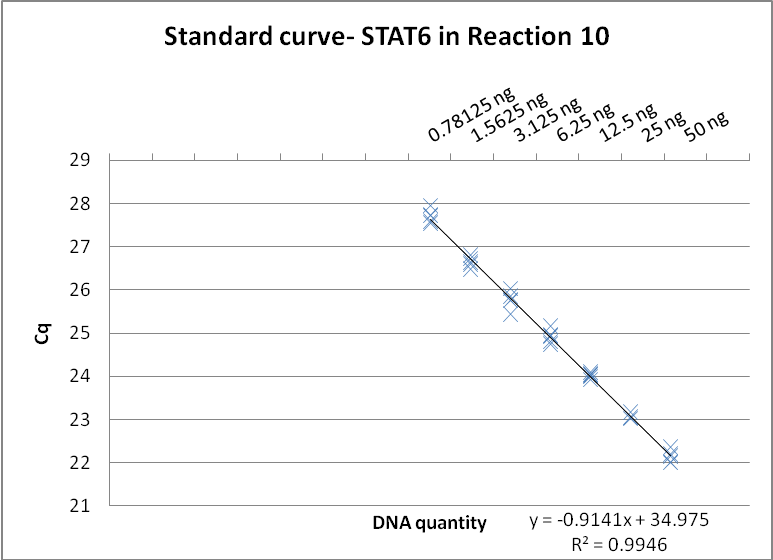


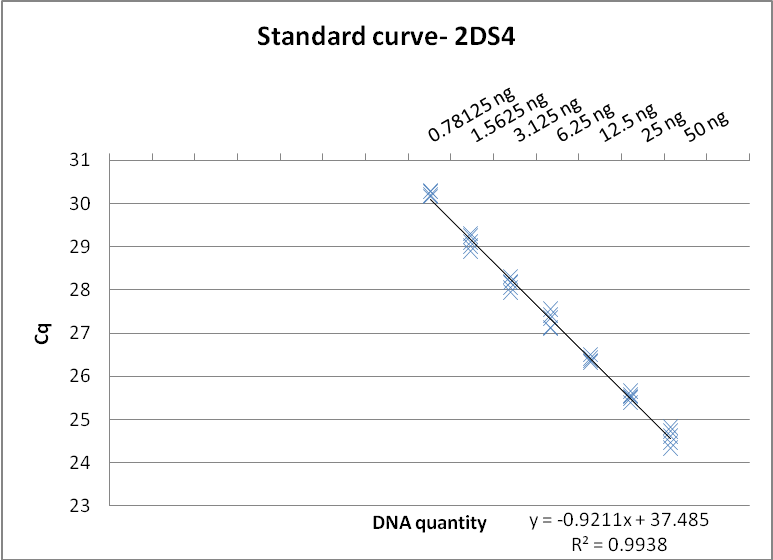


# **Supplementary Figure 3:** **Analysis of assay performance using standard curves**

The overall performance of each reaction was tested using standard curves from a verified positive DNA sample carrying the gene being tested. A two-fold dilution series from 50ng to 0.78125ng per reaction and quadruplicate of each concentration was used to generate the standard curve. Otherwise, the PCR conditions were the same as above. Standard curve plots were generated by plotting PCR C_q_ value against the logarithmic value of template DNA quantity in each reaction. The final selection of primer sequences, probe sequences and their concentration in each reaction are listed in [Supplementary Tables 1](#_Supplementary_Table_1:), [2](#_Supplementary_Table_2:) and [3](#_Supplementary_Table_3:) respectively. The slopes of the standard curve were used to calculate the efficiency of the PCR reactions (see [Supplementary Table 4](#_Supplementary_Table_4:) for more details). The y-intercept gives indication of the sensitivity of the assay. R^2^ is the square of the coefficient of regression; the value indicates how good the line fits the data (see [Supplementary Table 4](#_Supplementary_Table_4:) for more details).


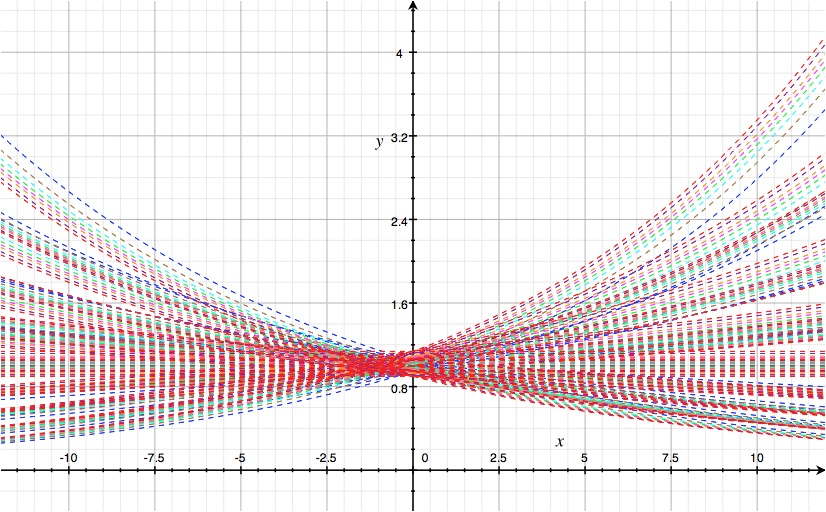

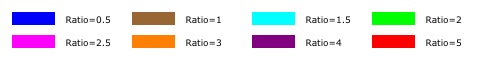


**Supplementary Figure 4: Divergence of calculated copy number without efficiency correction and true copy number**

Efficiency of target and reference assay is between 0.9 and 1.1. The ratio is the fold difference of target assay in test sample and calibrator, which is given the value of 0.5, 1, 1.5, 2, 2.5, 3, 4, and 5. x-axis: ∆C_q_ of reference gene between sample and calibrator. y-axis: fold change of calculated and true copy number.


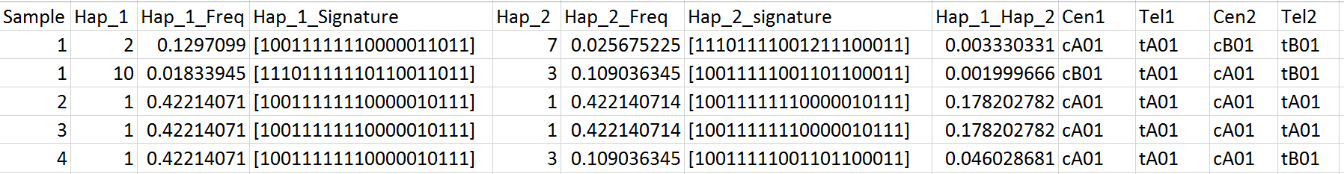

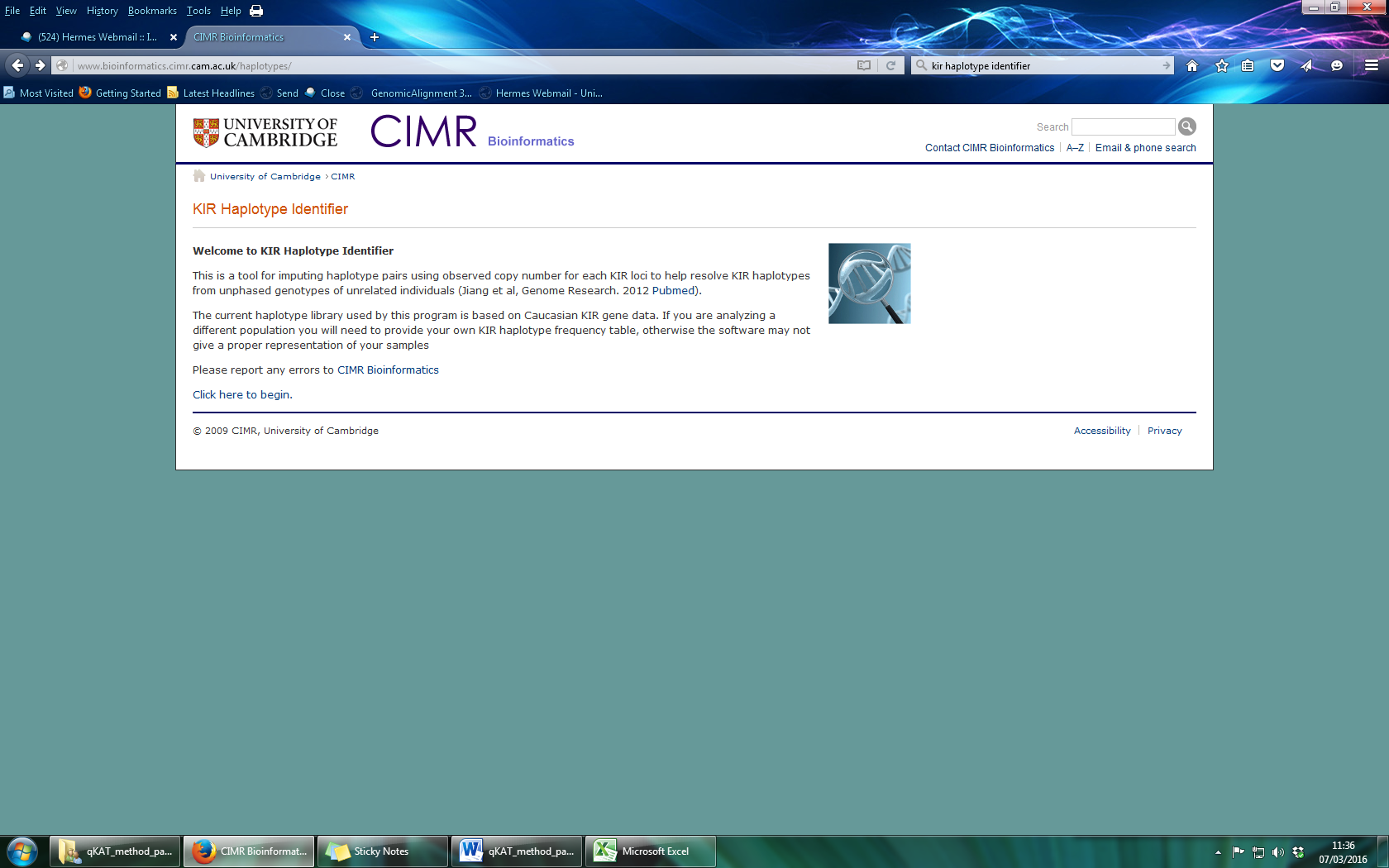


# **Supplementary Figure 5: The KIR Haplotype Identifier Tool**

Top: The KIR Haplotype Identifier Tool homepage. Bottom: Representative example of output (Haplotype Results file) from the tool. The output shows the possible combinations of haplotypes for each sample based on the gene content of all haplotypes supplied in the haplotype file. The file lists all possible haplotype pairs for each sample, each haplotypes frequency (from the haplotype file) and the predicted combined frequency of each haplotype pair. Haplotype signature and annotation according to centromeric motif and telomeric motif structure is also given. The output shows that Sample 1 carries either haplotype 2 and haplotype 7 or haplotype 10 and haplotype 3. The haplotype combination for Sample 2 (haplotype 1 and haplotype 1), Sample 3 (haplotype 1 and haplotype 1) and Sample 4 are unambiguous (haplotype 1 and haplotype 3).


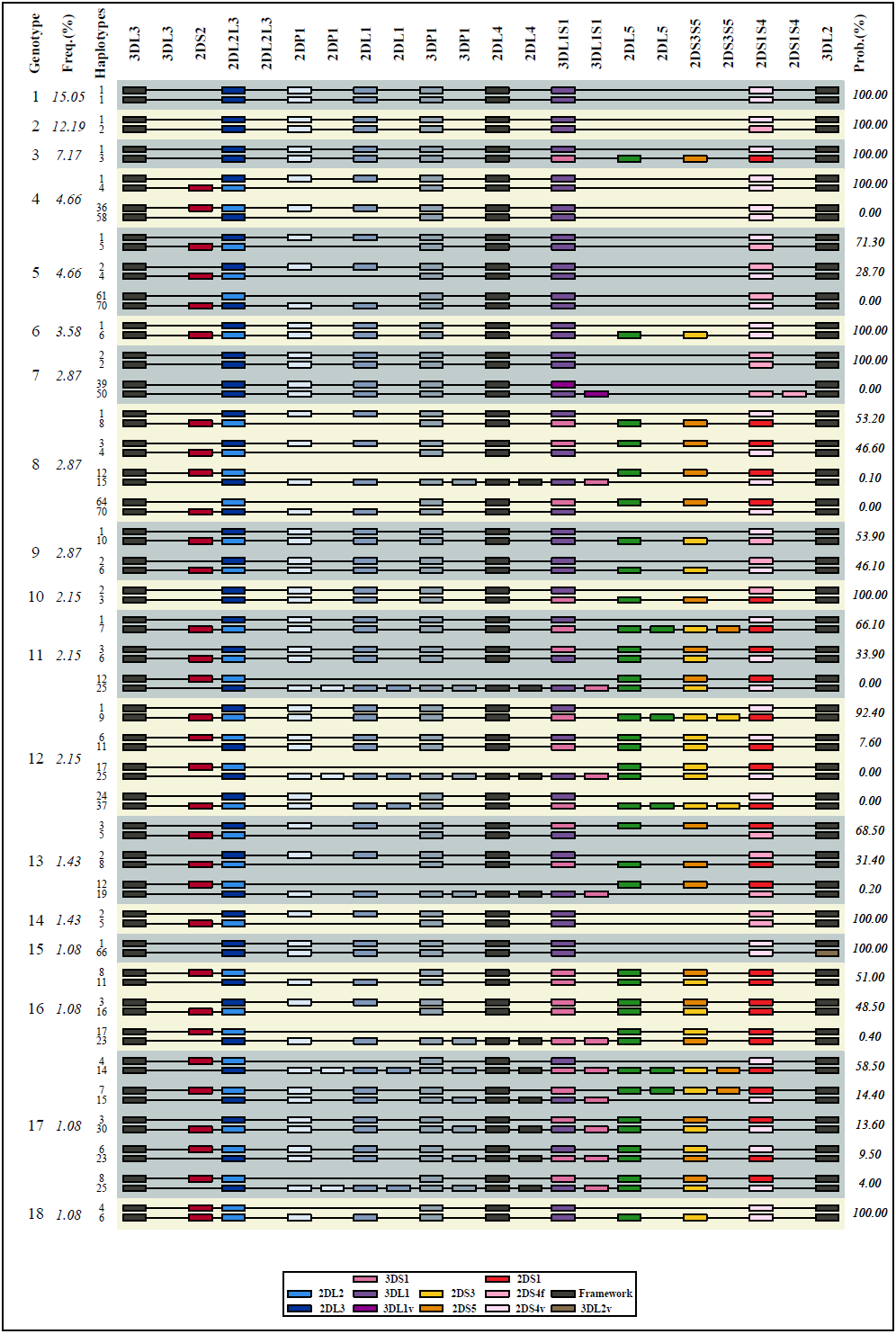


# **Supplementary Figure 6: An example of the output from the KIR Haplotype Resolution Drawing Tool**

*KIR* copy number data from a previously published study ([Jiang et al. 2012](#_ENREF_5)) was used as input. Shown are the observed *KIR* genotypes observed in the study panel and their possible haplotype resolutions based on confirmed haplotypes observed in European populations. The genotypes are ordered by the frequency in the sample. For each genotype, all possible haplotype combinations are listed with probability computed from the estimated haplotype frequencies. Genotypes not solvable with known haplotypes are marked with '?' (not shown in this figure as all listed genotypes were solved). The colours representing *KIR* genes are similar to Figure 4 of Jiang et al. (2012). Genes suffixed with 'v' have parts altered or deleted including fused genes resulting from NAHR or a novel allele (see ([Jiang et al. 2012](#_ENREF_5))).


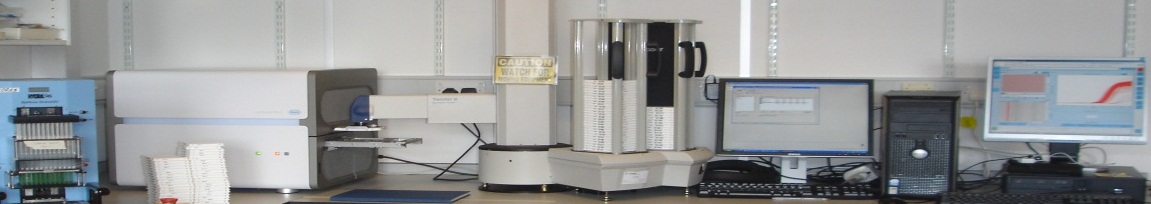


# **Supplementary Figure 7: Instruments for the *KIR* copy number assay**

The instrument used for semi-automated *KIR* copy number assay. From left to right: Matrix Hydra II (Thermo Scientific), LightCycler 480 Real-Time PCR System with 384-well Thermal Block Cycler (Roche Applied Science), Twister II Plate Handler (Caliper) with MéCour Thermal Plate Stacker (MéCour), Twister II control computer (Dell), LightCycler 480 control computer (HP).

Initial error rate:

# **Supplementary Figure 8: Probability of genotype calling error decreases as number of offspring increase**

Different series indicate the original probability of error when trios were used. When additional offspring are used:

$$P_{new}=P_{ori}{(1-P_{ori})}^{n-1}$$

Equation 14

P_new_: the new probability; P_ori_: original probability with trios; n: number of offspring.

Initial probability:

# **Supplementary Figure 9: Probability of solving the haplotype phase increases as number of offspring increase**

Different series indicate the original probability (denoted by P_ori_) when trios were used. When additional offspring (denoted by n) were considered, the new probability (denoted by P_new_) can be calculated using the following equation.

$$P_{new}=1-{(1-P_{ori})}^{n}$$

Equation 13

Due to the complexities inherent in copy number determination and the limited number of offspring for analysis in some family material, it is not possible to determine all of the chromosome-specific copy number phases. However, it is still valuable to set up a default haplotype code in order to aid subsequent analysis. The default phase should be the one with higher frequency and could be determined using the equations listed in [Supplementary Table 8](#_Supplementary_Table_9:). However, as the default phase is set to the most frequent one, this procedure will inevitably cause the less frequent phase to be underrepresented in the results. Although there is no way of resolving all of the ambiguities, the probability of this type of error can be estimated, using equations listed in [Supplementary Table 12](#_Supplementary_Table_10:).

| **Reactions** | **Slope** | **R^2^** | **Efficiency (%)** |
| --- | --- | --- | --- |
| 3DP1 | -1.0442 | 0.9948 | 94.2172 |
| STAT6 in reaction 1 | -1.0343 | 0.9927 | 95.4551 |
| 2DL2 | -1.0055 | 0.9929 | 99.2431 |
| 2DS2 | -0.9751 | 0.9919 | 103.5715 |
| STAT6 in reaction 2 | -1.0378 | 0.9966 | 95.0139 |
| 2DL3 | -1.0350 | 0.9929 | 95.3666 |
| 3DL3 | -1.0481 | 0.9938 | 93.7381 |
| STAT6 in reaction 3 | -0.9850 | 0.9974 | 102.1223 |
| 2DS4Del | -1.0174 | 0.9967 | 97.6431 |
| 3DL1e4 | -0.9936 | 0.9908 | 100.8949 |
| STAT6 in reaction 4 | -1.0111 | 0.9928 | 98.4839 |
| 3DL1e9 | -1.0075 | 0.9947 | 98.9707 |
| 3DS1 | -0.9825 | 0.9940 | 102.4845 |
| STAT6 in reaction 5 | -0.9433 | 0.9952 | 108.5088 |
| 2DL4 | -0.9562 | 0.9906 | 106.4520 |
| 2DL1 | -1.0731 | 0.9984 | 90.7760 |
| STAT6 in reaction 6 | -1.0269 | 0.9980 | 96.4013 |
| 2DP1 | -1.0524 | 0.9966 | 93.2153 |
| 2DS1 | -0.9951 | 0.9872 | 100.6838 |
| STAT6 in reaction 7 | -1.0456 | 0.9939 | 94.0447 |
| 2DL5 | -1.0367 | 0.9932 | 95.1521 |
| 2DS3 | -1.0347 | 0.9900 | 95.4045 |
| STAT6 in reaction 8 | -1.0224 | 0.9930 | 96.9857 |
| 3DL2e9 | -1.0293 | 0.9961 | 96.0925 |
| 3DL2e4 | -1.0383 | 0.9956 | 97.4756 |
| STAT6 in reaction 9 | -0.9988 | 0.9985 | 100.0833 |
| 2DS4FL | -1.0512 | 0.9962 | 96.6803 |
| 2DS5 | -0.9136 | 0.9940 | 106.7748 |
| STAT6 in reaction 10 | -0.9141 | 0.9946 | 106.7305 |
| 2DS4 | -0.9211 | 0.9938 | 106.1172 |

# **Supplementary Table 1: PCR efficiency of each reaction**

The PCR efficiencies were calculated from the slope value generated from [Supplementary Figure 3](#_Supplementary_Figure_3:) using the equation below.

Efficiency = [2^(-1 / slope)^] – 1

**Equation 1**

Slope and R^2^ were calculated using Excel’s linear regression function. In this assay, a two-fold series dilution was used, so a calculated slope between −1.07991and −0.93424 is equivalent to 90-110% reaction efficiency, which is generally acceptable in most occasions for accurate quantification.

In this assay, four replicates were used for each concentration in the standard curve. The R^2^ is the square of correlation coefficient and it is a measure of how accurate future values could be predicted by the model. In theory the value should be 1. However, this value is influenced by experimental factors such as pipetting or well-to-well variation from the real-time PCR instrument. An empirical value for R^2^ >0.985 is generally accepted.

# **Supplementary Note: PCR efficiency and relative quantification**

Based on the exponential amplification of PCR, the amount of PCR product at the cycle of quantification is calculated by:

$$N_{Cq}=N_{0}{C(1+E)}^{Cq}$$

**Equation 2**

Where N_Cq_ is the number of amplicons at the the threshold cycle, N_0_ is the initial number of chromosomes containing target DNA in the sample; C is the copy number of target gene per chromosome; E is the efficiency of the PCR assay and C_q_ is the cycle of quantification.

The equations for the sample and calibrator in the target and reference assay can be written in a similar way. For sample in target assay:

|  | $N_{target\_sample}=N_{0\_sample}C_{target\_sample}{(1+E_{target})}^{{Cq}_{target\_sample}}$ |  |
| --- | --- | --- |

**Equation 3**

For calibrator in target assay:

|  | $N_{target\_calibrator}=N_{0\_calibrator}C_{target\_calibrator}{(1+E_{target})}^{{Cq}_{target\_calibrator}}$ |  |
| --- | --- | --- |

**Equation 4**

For sample in reference assay:

|  | $N_{ref\_sample}=N_{0\_sample}C_{ref\_sample}{(1+E_{ref})}^{{Cq}_{ref\_sample}}$ |  |
| --- | --- | --- |

**Equation 5**

For calibrator in reference assay:

|  | $N_{ref\_calibrator}=N_{0\_calibrator}C_{ref\_calibrator}{(1+E_{ref})}^{{Cq}_{ref\_calibrator}}$ |  |
| --- | --- | --- |

**Equation 6**

There should be the same number of PCR amplicons at the threshold cycle for the same PCR assay. Moreover, different samples should have the same copy number of reference assay. Thus, from Equation 5 and Equation 6:

|  | $\frac{N_{0\_sample}}{N_{0\_calibrator}}=\frac{{(1+E_{ref})}^{{Cq}_{ref\_calibrator}}}{{(1+E_{ref})}^{{Cq}_{ref\_sample}}}={(1+E_{ref})}^{{\Delta Cq}_{ref\_(calibrator-sample)}}$ |  |
| --- | --- | --- |

**Equation 7**

Similarly, Equation 3 and Equation 4 can be rewritten for the target assay:

|  | $\frac{C_{target\_sample}}{C_{target\_calibrator}}=\frac{N_{0\_calibrator}\left( 1+E_{target} \right)^{{Cq}_{target\_calibrator}}}{N_{0\_sample}\left( 1+E_{target} \right)^{{Cq}_{target\_sample}}}$  =${(1+E_{ref})}^{{\Delta Cq}_{ref}\_(sample-calibrator)}{(1+E_{target})}^{{-\Delta Cq}_{target\_(sample-calibrator)}}$ |  |
| --- | --- | --- |

**Equation 8**

If the efficiency of both target and reference assay are roughly close to 1, then Equation 8 can be rewritten as:

|  | $\frac{C_{target\_sample}}{C_{target\_calibrator}}=2^{-[{(Cq}_{target} - {Cq}_{ref})Sample -{(Cq}_{target} - {Cq}_{ref})Calibrator]}$ |  |
| --- | --- | --- |
| or | $\frac{C_{target\_sample}}{C_{target\_calibrator}}= 2^{-{\Delta\Delta C}_{q}}$ |  |

**Equation 9**

The copy number of the calibrator in the target assay is always known. Therefore, the copy number of target gene in test sample can be easily calculated. However, instead of 100%, PCR efficiency usually varies from assay to assay. Generally efficiency ranges from 90%-110% is accepted in relative quantification ([Bustin 2004](#_ENREF_2)). Equation 9 is widely used in the copy number calculation when effeciency is not corrected. From Equation 8 and Equation 9, the difference between calculated copy number and true copy number can be shown using the following equations:

|  | $\frac{Calculated Copy number}{True copy number}=\frac{2^{\Delta{Cq}_{ref}}2^{-\Delta{Cq}_{target}}}{{(1+E_{ref})}^{\Delta{Cq}_{ref}}\left( 1+E_{target} \right)^{-\Delta{Cq}_{target}}}$ |  |
| --- | --- | --- |

**Equation 10**

From Equation 8, $\frac{C_{target\_sample}}{C_{target\_calibrator}}$ is the ratio of copy number between sample and calibrator in the target assay. Normally, the copy number of calibrator sample in the target assay is one or two. Then for target assay with low copy number repeats, the ratio would be 0.5, 1, 1.5, 2, 2.5, 3, 4, 5 and so on. Therefore, from Equation 8 , ΔCq_ref_(sample-calibrator)_ can be calculated as follows:

|  | ${\Delta Cq}_{target}=\frac{lg\left( ratio \right)+{\Delta Cq}_{ref}lg(1+E_{ref})}{lg(1+E_{target})}$ |  |
| --- | --- | --- |

**Equation 11**

Then Equation 10 can be transformed as follows:

|  | $\frac{Calculated CN}{True CN}={{(\frac{2}{1+E_{ref}})}^{-\Delta{Cq}_{ref}}(\frac{2}{1+E_{target}})}^{\frac{lg\left( ratio \right)+{\Delta Cq}_{ref}lg(1+E_{ref})}{lg(1+E_{target})}}$ |  |
| --- | --- | --- |

**Equation 12**

| **Gene** | **Forward Primer (5´-3´)** | **Reverse Primer (5´-3´)** | **Probe (5´-3´)** |
| --- | --- | --- | --- |
| *LILRA1* | CCTCCCCAAGCCCACA | GGGAACTGGCCCTTCTTCA | TCCTGGAGACCCAGGAGTACCGTCTG |
| *LILRA2* | GCCAGGCTCTGTGATCAT | AACCCAGGATGCTGATTTG | AAGTCCTGTGACCCTCAGGTGTCAG |
| *LILRA3* | AAGGAAGGAGAAGATGAACACC | GTAGAGACCACACATAGGGAGC | CCATCTTCTCCGTGGGCC |
| *LILRA4* | CCTCACGGCTGGGACTG | TAGCCGTAGCATCTGAATGTACC | TTGAGGAAGGAGACCACAGGCTCTCC |
| *LILRA5* | TGGTGACCTCAGGAGAGAAC | AACAGGGCCTGGAACTG | ACGGCTGAGATTCGACAGGTTCAT |
| *LILRA6* | CTCACACGCCAAGGATTACA | GAACACCAGGACCAAGCCT | ATGCCCATGCGGATGAGATTC |
| *LILRB1* | GTGAACTCAGGAGGGAATGTA | GTCATAAGCATAGCACCTGTACC | CCATCTTCTCCGTGGGCC |
| *LILRB2* | GAGTCCCGTCACCCTCAGT | CAGGTGATGGATGGGATGT | TCTTGGATTACACGGATACGACCAGAGC |
| *LILRB3* | TGGGAAGATACCTGGAGGTTT | CAGATGTCCTGTGTTTGCTG | AGCAGCAGGACGAAGGCCAC |
| *LILRB4* | TGCTGTGTCAGTCACGGA | GGGGTGTGACAGCAGGTAGT | ATCAGAGCACGGAGCTCAGCAGC |
| *LILRB5* | CTGTGATAGCTCGGGGGAAG | GCTCCAGTGGGTTCTGTCTC | CCGTCTGGATAAGGAGGGACTCCCAT |
| *STAT6* | CCAGATGCCTACCATGGTGC | CCATCTGCACAGACCACTCC | CTGATTCCTCCATGAGCATGCAGCTT |

# **Supplementary Table 2: Primer and probe sequences used in *LILR* copy number assays**

Each assay includes one *LILR* target and one reference (*STAT6*) reaction. Probes for target genes were dual-labelled with the dye FAM and the quencher BHQ-1. STAT6 probe was labelled with DFO and BHQ-2. Primer concentrations 125-500nM. Probe concentration 150nM.

| **Gene** | **Primers** | **Direction** | **Sequence (5´-3´)** | **Length** | **Tm ^a^** | **GC%** | **Exon** | **Position ^b^** | **Amplicon (bp)** | **Alleles might miss** | **Reference** |
| --- | --- | --- | --- | --- | --- | --- | --- | --- | --- | --- | --- |
| ***3DL2e4*** | A1F | Forward | GCCCCTGCTGAAATCAGG | 18 | 52 | 61.1 | 4 | 399-416 | 179 | 3DL2*008, *021, *027, *038. |  |
|  | A1R | Reverse | CTGCAAGGACAGGCATCAA | 19 | 53 | 52.6 |  | 559-577 |  | 3DL2*048 |  |
| ***3DP1*** | A4F | Forward | GTCCCCTGGTGAAATCAGA | 19 | 49 | 52.6 | 4 | 398-416 | 112 | No |  |
|  | A5R | Reverse | GTGAGGCGCAAAGTGTCA | 18 | 52 | 55.6 |  | 492-509 |  | No |  |
| ***2DS2*** | A4F | Forward | GTCCCCTGGTGAAATCAGA | 19 | 49 | 52.6 | 4 | 398-416 | 111 | No |  |
|  | A6R | Reverse | TGAGGTGCAAAGTGTCCTTAT | 21 | 51 | 42.9 |  | 488-508 |  | No |  |
| ***3DL3 ^c^*** | A8Fa | Forward | GTGAAATCGGGAGAGACG | 18 | 50 | 55.6 | 4 | 406-423 | 139 | No |  |
|  | A8Fb | Forward | GGTGAAATCAGGAGAGACG | 19 | 50 | 52.6 |  | 405-423 |  | 3DL3*054, 3DL3*00905. |  |
|  | A8R | Reverse | AGTTGACCTGGGAACCCG | 18 | 51 | 61.1 |  | 526-543 |  | No |  |
| ***3DL1e4*** | B1F | Forward | CATCGGTCCCATGATGCT | 18 | 51 | 55.6 | 4 | 549-566 | 85 | 3DL1*006, 3DL1*054 | ([Vilches et al. 2007](#_ENREF_7)) |
|  | B1R | Reverse | GGGAGCTGACAACTGATAGG | 20 | 52 | 55 |  | 614-633 |  | 3DL1*00502 |  |
| ***3DS1*** | B2F | Forward | CATCGGTTCCATGATGCG | 18 | 51 | 55.6 | 4 | 549-566 | 85 | 3DS1*047; may pick up 3DL1*054. | ([Vilches et al. 2007](#_ENREF_7)) |
|  | B1R | Reverse | GGGAGCTGACAACTGATAGG | 20 | 52 | 55 |  | 614-633 |  | No |  |
| ***2DL1*** | B3F | Forward | TTCTCCATCAGTCGCATGAC | 20 | 52 | 50 | 4 | 544-563 | 96 | 2DL1*020 |  |
|  | B3R | Reverse | GTCACTGGGAGCTGACAC | 18 | 50 | 61.1 |  | 622-639 |  | 2DL1*023 |  |
| ***2DS1*** | B4F | Forward | TCTCCATCAGTCGCATGAA | 19 | 51 | 47.4 | 4 | 545-563 | 96 | 2DS1*001 | ([Vilches et al. 2007](#_ENREF_7)) |
|  | B4R | Reverse | GGTCACTGGGAGCTGAC | 17 | 49 | 64.7 |  | 624-640 |  | No | ([Vilches et al. 2007](#_ENREF_7)) |
| ***2DS3*** | B5F | Forward | CTCCATCGGTCGCATGAG | 18 | 53 | 61.1 | 4 | 546-563 | 96 | No |  |
|  | B5R | Reverse | GGGTCACTGGGAGCTGAA | 18 | 51 | 61.1 |  | 624-641 |  | No |  |
| ***2DS5*** | B6F2 | Forward | AGAGAGGGGACGTTTAACC | 19 | 50 | 52.6 | 4 | 475-493 | 173 | No | ([Vilches et al. 2007](#_ENREF_7)) |
|  | B6R3 | Reverse | TCCAGAGGGTCACTGGGC | 18 | 53 | 66.7 |  | 630-647 |  | 2DS5*003 |  |
| ***2DL4*** | C1F | Forward | GCAGTGCCCAGCATCAAT | 18 | 52 | 55.6 | 5 | 808-825 | 83 | No |  |
|  | C1R | Reverse | CCGAAGCATCTGTAGGTCT | 19 | 52 | 52.6 |  | 872-890 |  | 2DL4*018, 2DL4*019 |  |
| ***2DL2*** | 2DL2F4 | Forward | GAGGTGGAGGCCCATGAAT | 19 | 52 | 57.9 | 5 | 778-796 | 151 | 2DL2*009; 782G changed to A. | ([Ashouri et al. 2009](#_ENREF_1)) |
|  | C3R2 | Reverse | TCGAGTTTGACCACTCGTAT | 20 | 51 | 45 |  | 909-928 |  | No | ([Martin and Carrington 2008](#_ENREF_6)) |
| ***2DS4*** | C5F | Forward | TCCCTGCAGTGCGCAGC | 17 | 57 | 70.6 | 5 | 803-819 | 120 | No |  |
|  | C5R | Reverse | TTGACCACTCGTAGGGAGC | 19 | 52 | 57.9 |  | 904-922 |  | 2DS4*013 | ([Ashouri et al. 2009](#_ENREF_1)) |
| ***(Continued)*** | | | | | | | | | | | |

| ***(Continued)*** | | | | | | | | | | | |
| --- | --- | --- | --- | --- | --- | --- | --- | --- | --- | --- | --- |
| **Gene** | **Primers** | **Direction** | **Sequence (5´-3´)** | **Length** | **Tm ^a^** | **GC%** | **Exon** | **Position ^b^** | **Amplicon (bp)** | **Alleles might miss** | **Reference** |
| ***2DS4Del*** | 2DS4Del | Forward | CCTTGTCCTGCAGCTCCAT | 19 | 54 | 57.9 | 5 | 750-768 | 203 | No |  |
|  | 2DS4R2 | Reverse | TGACGGAAACAAGCAGTGGA | 20 | 53 | 50 |  | 933-952 |  | No |  |
| ***2DS4FL*** | 2DS4FL | Forward | CCGGAGCTCCTATGACATG | 19 | 53 | 57.9 | 5 | 744-762 | 209 | No |  |
|  | 2DS4R2 | Reverse | TGACGGAAACAAGCAGTGGA | 20 | 53 | 50 |  | 933-952 |  | No |  |
| ***2DL3*** | D1F | Forward | AGACCCTCAGGAGGTGA | 17 | 48 | 58.8 | 9 | 1180-1196 | 156 | No | ([Vilches et al. 2007](#_ENREF_7)) |
|  | D1R ^d^ | Reverse | CAGGAGACAACTTTGGATCA | 20 | 50 | 45 |  | 1316-1335 |  | 2DL3*010, 2DL3*017. | ([Vilches et al. 2007](#_ENREF_7)) |
| ***2DL5*** | D2F | Forward | CACTGCGTTTTCACACAGAC | 20 | 52 | 50 | 9 | 1214-1233 | 120 | 2DL5B*011 |  |
|  | D2R | Reverse | GGCAGGAGACAATGATCTT | 19 | 49 | 47.4 |  | 1315-1333 |  | No |  |
| ***2DP1*** | D3F | Forward | CCTCAGGAGGTGACATACGT | 20 | 53 | 55 | 9 | 1184-1203 | 121 | No |  |
|  | D3R | Reverse | TTGGAAGTTCCGTGTACACT | 20 | 50 | 45 |  | 1285-1304 |  | No |  |
| ***3DL1e9*** | D4F | Forward | CACAGTTGGATCACTGCGT | 19 | 52 | 52.6 | 9 | 1203-1221 | 93 | 3DL1*061, 3DL1*068 |  |
|  | D4R2 ^e^ | Reverse | CCGTGTACAAGATGGTATCTGTA | 23 | 53 | 43.5 |  | 1273-1295 |  |  | ([Vilches et al. 2007](#_ENREF_7)) |
| ***3DL2e9*** | D4F | Forward | CACAGTTGGATCACTGCGT | 19 | 52 | 52.6 | 9 | 1203-1221 | 156 | No |  |
|  | D5R | Reverse | GACCTGACTGTGGTGCTCG | 19 | 54 | 63.2 |  | 1340-1358 |  | No |  |
| ***STAT6*** | STAT6F | Forward | CCAGATGCCTACCATGGTGC | 20 | 54 | 60 |  |  | 129 |  | ([Degenhardt et al. 2009](#_ENREF_3)) |
|  | STAT6R | Reverse | CCATCTGCACAGACCACTCC | 20 | 54 | 60 |  |  |  |  |  |

# **Supplementary Table 3: Primers used in *KIR* copy number assays**

a Primer Tm value was calculated using nearest neighbor method.

b Primer position numbering is based on coding sequence from IPD - KIR Database. (Release 2.4.0, 15 April 2011. http://www.ebi.ac.uk/ipd/kir/)

c A8Fa and A8Fb used together at the same concentration as 3DL3 forward primer.

d Since Release 2.2.0, 2DL3 sequences were truncated from position 1319 to position 1342, as sequence is not part of CDS. However, the ability of oligo binding to the Genomic sequence is not affected.

e Primer was designed to miss the 3DL1/3DL2 fusion allele: 3DL1*059, 3DL1*060, 3DL1*061, 3DL1*064 and 3DL1*065.

e4 = exon4 and e9 = exon9

In the few instances in which a rare SNP lies within an annealing site for a primer and may therefore disrupt binding, the corresponding allele designation is given.

With the exception of *3DL1* and *3DL2* (which have two assays anyway) all “missed” alleles have not been seen in any population of European-ancestry at time of writing ([Gonzalez-Galarza et al. 2015](#_ENREF_4)).

| **Name** | **Direction** | **5´ modification** | **3´ modification** | **Sequence** | **Length** | **Tm ^a^** | **GC%** | **Exon** | **Position ^b^** |
| --- | --- | --- | --- | --- | --- | --- | --- | --- | --- |
| **P4a** | Sense | FAM | BHQ-1 | TCATCCTGCAATGTTGGTCAGATGTCA | 27 | 60 | 44.4 | 4 | 425-451 |
|  |  |  |  |  |  |  |  |  |  |
| **P4b** | Antisense | FAM | BHQ-1 | AACAGAACCGTAGCATCTGTAGGTCCCT | 28 | 62 | 50 | 4 | 576-603 |
|  |  |  |  |  |  |  |  |  |  |
| **P5b** | Sense | Cy5 | BHQ-2 | AACATTCCAGGCCGACTTTCCTCTG | 25 | 60 | 52 | 5 | 828-852 |
|  |  |  |  |  |  |  |  |  |  |
| **P5b-2DL4** | Sense | Cy5 | BHQ-2 | AACATTCCAGGCCGACTTCCCTCTG | 25 | 61 | 56 | 5 | 828-852 |
|  |  |  |  |  |  |  |  |  |  |
| **P9** | Sense | Cy5 | BHQ-2 | CCCTTCTCAGAGGCCCAAGACACC | 24 | 60 | 62.5 | 9 | 1246-1269 |
|  |  |  |  |  |  |  |  |  |  |
| **PSTAT6 ^c^** |  | DFO | BHQ-2 | CTGATTCCTCCATGAGCATGCAGCTT | 26 | 62 | 50 |  |  |

# **Supplementary Table 4: Probes used in *KIR* copy number assays**

a Primer Tm value was calculated using nearest neighbor method.

b Primer position numbering is based on coding sequence from IPD - KIR Database. (Release 2.4.0, 15 April 2011. http://www.ebi.ac.uk/ipd/kir/)

c Previously published 36.

In the few instances in which a rare SNP lies within an annealing site for a probe and may therefore disrupt binding, the corresponding allele designation is given.

| **Assay** | **Genes** | **Forward Primers** | **Concentra-tion (nM)** | **Reverse Primers** | **Concentra-tion (nM)** | **Probes** | **Concentra-tion (nM)** | | | **Alleles may miss by probe** | |
| --- | --- | --- | --- | --- | --- | --- | --- | --- | --- | --- | --- |
| **No 1** | ***3DP1*** | A4F | 250 | A5R | 250 | P4a | | 150 | No | |  |
|  | ***2DL2*** | 2DL2F4 | 400 | C3R2 | 600 | P5b | | 150 | No | |  |
|  | ***STAT6*** | STAT6F | 200 | STAT6R | 200 | PSTAT6 | | 150 |  | |  |
|  |  |  |  |  |  |  | |  |  | |  |
| **No 2** | ***2DS2*** | A4F | 400 | A6R | 400 | P4a | | 200 | No | |  |
|  | ***2DL3*** | D1F | 400 | D1R | 400 | P9 | | 150 | 2DL3*01201 | |  |
|  | ***STAT6*** | STAT6F | 200 | STAT6R | 200 | PSTAT6 | | 150 |  | |  |
|  |  |  |  |  |  |  | |  |  | |  |
| **No 3** | ***3DL3*** | A8F | 500 | A8R | 500 | P4a | | 150 | No | |  |
|  | ***2DS4Del*** | 2DS4Del | 250 | 2DS4R2 | 250 | P5b | | 150 | 2DS4*009 | |  |
|  | ***STAT6*** | STAT6F | 200 | STAT6R | 200 | PSTAT6 | | 150 |  | |  |
|  |  |  |  |  |  |  | |  |  | |  |
| **No 4** | ***3DL1e4*** | B1F | 250 | B1R | 125 | P4b | | 150 | 3DL1*01503, *056 | |  |
|  | ***3DL1e9*** | D4F | 250 | D4R2 | 500 | P9 | | 150 | No (Designed to miss 3DL1*060) | |  |
|  | ***STAT6*** | STAT6F | 200 | STAT6R | 200 | PSTAT6 | | 150 |  | |  |
|  |  |  |  |  |  |  | |  |  | |  |
| **No 5** | ***3DS1*** | B2F | 250 | B1R | 250 | P4b | | 150 | No | |  |
|  | ***2DL4*** | C1F | 200 | C1R | 200 | P5b-2DL4 | | 150 | 2DL4*00901, *00902, *021 | |  |
|  | ***STAT6*** | STAT6F | 200 | STAT6R | 200 | PSTAT6 | | 150 |  | |  |
|  |  |  |  |  |  |  | |  |  | |  |
| **No 6** | ***2DL1*** | B3F | 500 | B3R | 125 | P4b | | 150 | No | |  |
|  | ***2DP1*** | D3F | 250 | D3R | 500 | P9 | | 150 | No | |  |
|  | ***STAT6*** | STAT6F | 200 | STAT6R | 200 | PSTAT6 | | 150 |  | |  |
|  |  |  |  |  |  |  | |  |  | |  |
| **No 7** | ***2DS1*** | B4F | 500 | B4R | 250 | P4b | | 150 | No | |  |
|  | ***2DL5*** | D2F | 500 | D2R | 500 | P9 | | 150 | No | |  |
|  | ***STAT6*** | STAT6F | 200 | STAT6R | 200 | PSTAT6 | | 150 |  | |  |
|  |  |  |  |  |  |  | |  |  | |  |
| **No 8** | ***2DS3*** | B5F | 250 | B5R | 250 | P4b | | 150 | No | |  |
|  | ***3DL2e9*** | D4F | 250 | D5R | 125 | P9 | | 150 | No | |  |
|  | ***STAT6*** | STAT6F | 200 | STAT6R | 200 | PSTAT6 | | 150 |  | |  |
|  |  |  |  |  |  |  | |  |  | |  |
| **No 9** | ***3DL2e4*** | A1F | 200 | A1R | 200 | P4a | | 150 | 3DL2*00502, *01102, *01302, *030, *051, *056 | |  |
|  | ***2DS4FL*** | 2DS4FL | 250 | 2DS4R2 | 500 | P5b | | 150 | 2DS4*00103 | |  |
|  | ***STAT6*** | STAT6F | 200 | STAT6R | 200 | PSTAT6 | | 150 |  | |  |
|  |  |  |  |  |  |  | |  |  | |  |
| **No 10** | ***2DS5*** | B6F2 | 200 | B6R3 | 200 | P4b | | 150 | No | |  |
|  | ***2DS4*** | C5F | 250 | C5R | 250 | P5b | | 150 | 2DS4*009, 00103 | |  |
|  | ***STAT6*** | STAT6F | 200 | STAT6R | 200 | PSTAT6 | | 150 |  | |  |

# **Supplementary Table 5: Primer and probe combinations used in *KIR* assays**

Each assay includes two *KIR* targets and one reference reaction. In total, ten assays were used to detect the copy number of all *KIR* loci. With the exception of *KIR3DL1* and *KIR3DL2* (which have two assays anyway) all “missed” alleles have not been seen in any population of European-ancestry at time of writing ([Gonzalez-Galarza et al. 2015](#_ENREF_4)).

| **Copy number difference** | **Fold change** | **C_q_ difference** | **SD to quantify 99.6% cases** | **SD to quantify 95% cases** |
| --- | --- | --- | --- | --- |
| 1 to 2 | 2 | 1 | < 0.1667 | < 0.25 |
| 2 to 3 | 1.5 | 0.585 | < 0.0975 | < 0.1463 |
| 3 to 4 | 1.3333 | 0.4151 | < 0.0692 | < 0.1038 |
| 4 to 5 | 1.25 | 0.3219 | < 0.0537 | < 0.0805 |
| 5 to 6 | 1.2 | 0.263 | < 0.0438 | < 0.0658 |
| 6 to7 | 1.1667 | 0.2224 | < 0.0371 | < 0.0556 |

# **Supplementary Table 6: Cq difference between different copy numbers and standard deviations required to distinguish them**

| **Family member** | **CEPH 1347** | **CEPH 1332** | **CEPH 1416** |
| --- | --- | --- | --- |
| Paternal grandfather | 2 | 3 | 2 |
| Paternal grandmother | 2 | 3 | 2 |
| Maternal grandfather | 3 |  | 2 |
| Maternal grandmother | 4 | 2 | 2 |
| Father | 2 | 3 | 2 |
| Mother | 2 | 2 | 3 |

# **Supplementary Table 7: *LILRA6* copy number in CEPH family samples**

| Gene | Predicted copy number | Number of values | Mean | Std. Deviation | Std. Error |  |
| --- | --- | --- | --- | --- | --- | --- |
|  |  |  |  |  |  |  |
| *2DL2* | 0 copy | 873 | 0.01488 | 0.02706 | 0.0009502 |  |
|  | 1 copy | 664 | 0.9973 | 0.07539 | 0.002866 |  |
|  | 2 copies | 156 | 1.897 | 0.1082 | 0.008665 |  |
|  | 3 copies | 4 | 2.835 | 0.1411 | 0.07053 |  |
| *3DP1* | 1 copy | 32 | 0.9852 | 0.08358 | 0.01501 |  |
|  | 2 copies | 1590 | 1.972 | 0.1131 | 0.002801 |  |
|  | 3 copies | 76 | 2.938 | 0.1561 | 0.01735 |  |
| *2DL3* | 0 copy | 167 | 0 | 0 | 0 |  |
|  | 1 copy | 682 | 0.9879 | 0.07558 | 0.002869 |  |
|  | 2 copies | 846 | 1.986 | 0.1431 | 0.004873 |  |
|  | 3 copies | 4 | 2.985 | 0.213 | 0.1065 |  |
| *2DS2* | 0 copy | 859 | 0.003861 | 0.02728 | 0.0009206 |  |
|  | 1 copy | 666 | 1.007 | 0.07913 | 0.003046 |  |
|  | 2 copies | 166 | 1.859 | 0.1354 | 0.009985 |  |
| *2DS4Del* | 0 copy | 342 | 0.000262 | 0.003362 | 0.0001813 |  |
|  | 1 copy | 807 | 1.024 | 0.08923 | 0.003062 |  |
|  | 2 copies | 550 | 1.884 | 0.1214 | 0.005132 |  |
| *3DL3* | 1 copy | 4 | 1.008 | 0.0957 | 0.04785 |  |
|  | 2 copies | 1692 | 1.968 | 0.1361 | 0.003266 |  |
|  | 3 copies | 5 | 2.992 | 0.06723 | 0.03007 |  |
| *3DL1e9* | 0 copy | 84 | 0.001608 | 0.01298 | 0.001086 |  |
|  | 1 copy | 606 | 1.001 | 0.09675 | 0.003879 |  |
|  | 2 copies | 1009 | 1.975 | 0.1015 | 0.003143 |  |
|  | 3 copies | 1 | 2.55 |  |  |  |
| *3DL1e4* | 0 copy | 83 | 6.94E-05 | 0.000833 | 6.944E-05 |  |
|  | 1 copy | 594 | 0.9993 | 0.08785 | 0.003569 |  |
|  | 2 copies | 1012 | 1.971 | 0.1199 | 0.003709 |  |
|  | 3 copies | 4 | 2.85 | 0.1493 | 0.07467 |  |
| *2DL4* | 1 copy | 33 | 1.008 | 0.2807 | 0.03455 |  |
|  | 2 copies | 1580 | 1.981 | 0.1977 | 0.004921 |  |
|  | 3 copies | 75 | 3.222 | 0.2034 | 0.02303 |  |
| *3DS1* | 0 copy | 1010 | 0.000528 | 0.00466 | 0.0001444 |  |
|  | 1 copy | 593 | 0.9933 | 0.09671 | 0.003942 |  |
|  | 2 copies | 101 | 1.965 | 0.1145 | 0.01117 |  |
|  | 3 copies | 4 | 2.973 | 0.04856 | 0.02428 |  |
| *2DP1* | 0 copy | 48 | 0.000208 | 0.001443 | 0.0002083 |  |
|  | 1 copy | 446 | 1.017 | 0.04951 | 0.002352 |  |
|  | 2 copies | 1189 | 1.973 | 0.09026 | 0.002662 |  |
|  | 3 copies | 12 | 2.83 | 0.1355 | 0.04284 |  |
| *2DL1* | 0 copy | 50 | 0.002245 | 0.006851 | 0.0009787 |  |
|  | 1 copy | 456 | 0.9996 | 0.06964 | 0.003265 |  |
|  | 2 copies | 1178 | 1.98 | 0.09868 | 0.002926 |  |
|  | 3 copies | 12 | 2.894 | 0.2324 | 0.07007 |  |
| *(Continued)* | | | | | | |

| *(Continued)* | | | | | | |
| --- | --- | --- | --- | --- | --- | --- |
| Gene | **Predicted copy number** | **Number of values** | **Mean** | **Std. Deviation** | **Std. Error** |  |
|  |  |  |  |  |  |  |
| *2DL5* | 0 copy | 833 | 0.000449 | 0.003799 | 0.0001289 |  |
|  | 1 copy | 575 | 0.9739 | 0.1412 | 0.005843 |  |
|  | 2 copies | 246 | 2.004 | 0.139 | 0.008789 |  |
|  | 3 copies | 42 | 2.97 | 0.158 | 0.02438 |  |
|  | 4 copies | 8 | 3.898 | 0.1574 | 0.05564 |  |
| *2DS1* | 0 copy | 1021 | 0.000236 | 0.002581 | 7.935E-05 |  |
|  | 1 copy | 599 | 0.9959 | 0.1226 | 0.004951 |  |
|  | 2 copies | 84 | 2.012 | 0.131 | 0.01429 |  |
| *3DL2e9* | 1 copy | 13 | 1.191 | 0.1451 | 0.03419 |  |
|  | 2 copies | 1686 | 1.984 | 0.1022 | 0.002458 |  |
| *2DS3* | 0 copy | 1235 | 0.000221 | 0.001935 | 5.435E-05 |  |
|  | 1 copy | 351 | 0.9938 | 0.1301 | 0.006809 |  |
|  | 2 copies | 95 | 1.985 | 0.08338 | 0.008555 |  |
|  | 3 copies | 13 | 2.86 | 0.1197 | 0.0332 |  |
|  | 4 copies | 1 | 4 | 0 | 0 |  |
| *2DS4FL* | 0 copy | 1057 | 0.000164 | 0.001405 | 4.237E-05 |  |
|  | 1 copy | 560 | 1.002 | 0.09588 | 0.004034 |  |
|  | 2 copies | 85 | 1.884 | 0.09727 | 0.01061 |  |
| *3DL2e4* | 1 copy | 17 | 0.9196 | 0.244 | 0.04981 |  |
|  | 2 copies | 1676 | 1.959 | 0.1784 | 0.004302 |  |
| *2DS4* | 0 copy | 81 | 0.000366 | 0.002457 | 0.0002713 |  |
|  | 1 copy | 600 | 1.009 | 0.05799 | 0.00235 |  |
|  | 2 copies | 1011 | 1.97 | 0.09344 | 0.002881 |  |
| *2DS5* | 0 copy | 1129 | 0.000658 | 0.009048 | 0.0002644 |  |
|  | 1 copy | 502 | 0.9853 | 0.09982 | 0.004442 |  |
|  | 2 copies | 66 | 2.015 | 0.1433 | 0.01738 |  |

# **Supplementary Table 8: Standard deviation in each cluster with the same assigned copy number**

Most of the standard deviations are below 0.15 (mean = 0.0950, standard deviation = 0.0687). The majority of the clusters fail the D'Agostino's K-squared test that means they are not normal distribution (the clusters of copy number are more compact than a normal distribution, allowing clear discrimination between different copy numbers).

| Total Copy number | Possible genotypes | Explanation |
| --- | --- | --- |
| 0 | 0/0 | Deletion on both chromosomes. |
| 1 | 0/1 | Deletion on one chromosome and one copy on the other. |
| 2 | 1/1;  0/2 | One copy on each chromosome;  Deletion and duplication on each chromosome. |
| 3 | 1/2;  0/3 | One copy on one chromosome and duplication on the other;  Deletion on one chromosome and three copies duplication on the other. |
| 4 | 2/2;  1/3;  0/4 | Two copies duplication on both chromosomes;  One copy on one chromosome and three copies duplication on the other;  Deletion on one chromosome and four copies duplication on the other. |

# **Supplementary Table 9:** **Inferring genotype from copy number**

There are genotype ambiguities when copy number is greater than one for any given locus. These explanations to infer *KIR* haplotypes using copy number information are based on one copy on each chromosome in a diploid genome. Copy numbers and the corresponding possible combinations of haplotypes are listed. Only total copy numbers up to four are considered because copy number greater than four is very rarely seen for the *KIR*. Previous studies investigating *KIR* genes inferred or deduced haplotypes using methods including family-based segregation analysis and allele typing [53-57], algorithms to predict haplotype frequency with [58, 59] or without pre-defined haplotypes [60] and full haplotype sequencing [61, 62]. Polymorphisms at both the gene copy number and allelic level of *KIR* loci make haplotype analysis challenging. Most of the previous studies investigating *KIR* gene profiles only focused on gene presence or absence information. The haplotypes identified by these methods are not necessarily definitive, since no information is available as to whether a certain *KIR* gene is present on one or both haplotypes. Allele typing and family-based segregation analysis potentially provide solutions to some of these problems. However, the accuracy depends on the number of SNPs used in allele typing and the size of family in segregation analysis. Bias can be introduced when studies rely on pre-defined haplotype patterns, which were characterized by the gene frequencies, LD information and consensuses among different studies [54].

Although spanning a region of ~150kb, the *KIR* loci show variable LD, adding another layer of difficulty for inferring the haplotypic phase in the segregation analysis. Furthermore, recently discovered extended and truncated haplotypes obscure the interpretation even further [3, 22, 26]. Segmental duplication and deletion caused by uneven crossover events indicate that multiple copies or zero copies of a certain gene could present on the same haplotype. Conventional methods have limited power to detect these kinds of variations. For example, a gene deletion is detectable only when the gene is missing from both chromosomes (zero copies). A gene duplication can, however, sometimes be revealed by allele typing if the alleles are different or at least different alleles are present on the same haplotype in family-based studies. In this study, a novel approach was used to infer the *KIR* haplotypes. Quantitative PCR and family-based segregation analysis provided gene dosage information and allowed phase determination of haplotypes. There have been a number of studies focused on the total copy numbers of genes in individuals and the association of such variations with diseases [63-65]. However, the copy number variations are actually independent for each chromosome in a diploid genome. For each locus, on the homologous chromosomes there could be a single copy on each or differing copy number. Therefore, determination of individual haplotypes is ambiguous as the experimentally-derived copy number is the total copy number from both chromosomes of each locus.

When pedigree data is not available, a convenient way to estimate the frequencies of haplotypic copy number is by using Hardy-Weinberg equilibrium (HWE)([Supplementary Table 10](#_Supplementary_Table_8:) and [Supplementary Table 11](#_Supplementary_Table_9:)).

| Copy number on each chromosome | 0 | 1 | 2 | 3 |
| --- | --- | --- | --- | --- |
| 0 | a^2^ | ab | ac | ad |
| 1 | ab | b^2^ | bc | bd |
| 2 | ac | bc | c^2^ |  |
| 3 | ad | bd |  |  |

# **Supplementary Table 10: Estimating haplotype frequency using Hardy-Weinberg equilibrium**

The number in the top row and first column indicates copy number on each haplotype; coloured cells indicate the total copy number: zero copy- orange, one copy- aqua, two copies- purple, three copies- olive green, four copies- red.

The frequencies of total copy numbers were experimentally-determined. Frequencies of total copy number are denoted by: zero copy = M, one copy = N, two copies = O, three copies = P, four copies = Q. Frequencies of haplotypic copy number are denoted by: zero copy = a, one copy = b, two copies = c, three copies = d. Assuming HWE, frequency of haplotype copy number can be calculated using the following equations:

M = a^2^; N = 2ab; O = 2ac + b^2^; P = 2ad + 2bc; Q = 2bd + c^2^

Although frequencies of haplotype phase can be estimated, the phase ambiguities still cannot be solved for each individual. Family-based haplotype inference can potentially provide solutions to this problem. The Mendelian inheritance in the families can help to determine the phase on each chromosome and consequently infer the haplotype.

| **Paternal haplotype** | | | | | | | |  |  |  |
| --- | --- | --- | --- | --- | --- | --- | --- | --- | --- | --- |
| **Zero** | **One** | **Two** | | **Three** | | **Four** | |  |  |  |
| 0/0 | 0/1 | 1/1 | 0/2 | 1/2 | 0/3 | 2/2 | 1/3 |  |  |  |
| 1 | 1 | 1 | | 1 | | 1 | | 0/0 | **Zero** | **Maternal haplotype** |
|  | 1 | 0.5(1-A) | | 0.5 | | 0.5-0.25C | | 0/1 | **One** |  |
|  | | 2A(1-A)+0.5(1-A)^2^ | | 0.5(1-B)(1-A) | | (0.5-0.25C)(1-A) | | 1/1 | **Two** |  |
|  |  |  |  |  |  |  |  | 0/2 |  |  |
|  | | | | 0.5(1-B^2^) | | 0.5(1-B)(1-C) | | 1/2 | **Three** |  |
|  |  |  |  |  |  |  |  | 0/3 |  |  |
|  | | | | | | 2C(1-C)+0.5(1-C)^2^ | | 2/2 | **Four** |  |
|  |  |  |  |  |  |  |  | 1/3 |  |  |

# **Supplementary Table 11: Probability to infer the haplotype-specific copy number using total copy number data from trios**

Inference of the haplotype-specific copy number in a trio (two parents and one child) when copy number phase is not known. Cells in blue and orange indicate paternal and maternal transmitted haplotype respectively. Possible genotypes are given below in the respective coloured cells. To simplify the calculation, 0/4 genotype (total copy number 4) is not included.

In some situations, two different genotypes could share the same total number. The percentage of each genotype in the ambiguities can be calculated using the following equations. The frequency of haplotype copy number is available from [Supplementary Table 8](#_Supplementary_Table_8:).

Total copy equals to two. Percentage of 1/1 genotype: $A = \frac{b^{2}}{b^{2}+2ac}$; 0/2 genotype: 1-A

Total copy equals to three. Percentage of 1/2 genotype: $B = \frac{bc}{bc+ad}$; 0/3 genotype: 1-B

Total copy equals to four. Percentage of 2/2 genotype: $C = \frac{c^{2}}{c^{2}+2bd}$; 1/3 genotype: 1-C

Only trios were used to infer the haplotypes. As the number of offspring increase, the probability to solve the phase problem will increase because there will be more transmission events available (see [Supplementary Figure 9](#_Supplementary_Figure_7:)). For example, if the initial probability of successful inference from trios is more than 0.2, with more than three siblings, the probability exceeds 0.5.

| **Paternal haplotype** | | | | | | | |  |  |  |
| --- | --- | --- | --- | --- | --- | --- | --- | --- | --- | --- |
| **Zero** | **One** | **Two** | | **Three** | | **Four** | |  |  |  |
| 0/0 | 0/1 | 1/1 | 0/2 | 1/2 | 0/3 | 2/2 | 1/3 |  |  |  |
| N/A | N/A | N/A | | N/A | | N/A | | 0/0 | **Zero** | **Maternal haplotype** |
|  | N/A | 0.5(1-A) | | 0.5(1-B) | | 0.5(1-C) | | 0/1 | **One** |  |
|  | | 0.5(1-A)^2^ | | 0.5(1-A) | | 0.5(1-C)(1-A) | | 1/1 | **Two** |  |
|  |  |  |  |  |  |  |  | 0/2 |  |  |
|  | | | | 0.5(1-B^2^) | | 0.5(1-C) | | 1/2 | **Three** |  |
|  |  |  |  |  |  |  |  | 0/3 |  |  |
|  | | | | | | 0.5(1-C)^2^ | | 2/2 | **Four** |  |
|  |  |  |  |  |  |  |  | 1/3 |  |  |

# **Supplementary Table 12: The probability of error when using a default haplotype code**

Cells in blue and orange indicate paternal and maternal transmitted haplotype respectively. Total copy number up to four is considered here. To simplify the calculation, 0/4 genotype (total copy number 4) is not included. In this Table, the 1/1, 0/2 and 2/2 genotypes are used as the default ones in the ambiguities. As the number of siblings helps to determine the phase (see [Supplementary Figure 9](#_Supplementary_Figure_7:)), the probability of error in phase calling drops as the number of siblings increases (see [Supplementary Figure 8](#_Supplementary_Figure_8:)). No matter what the original error rate is, with two offspring, the error rate will reduce to less than 0.25; with three offspring, the error rate will be less than 0.15; and with four offspring, the error rate will drop to 0.1. This is useful because with 3 to 4 siblings, the confidence to determine the haplotype phase is around 0.5 to 0.8 ([Supplementary Figure 9](#_Supplementary_Figure_7:)). However, assigned with the major phase, the chance of error is less than 0.15 ([Supplementary Figure 8](#_Supplementary_Figure_8:)). This method is valuable but the minor genotype may be underestimated.

| Test |
| --- |
| *KIR3DL3* CN = 2 |
| *KIR2DS2* CN = *KIR2DL2* CN |
| *KIR2DL2* CN + *KIR2DL3* CN = 2 |
| *KIR2DP1* CN = *KIR2DL1* CN |
| *KIR3DP1* CN = *KIR2DL4* CN |
| *KIR3DP1* CN + *KIR2DL4* CN = 4 |
| *KIR3DL1e4* CN = *KIR3DL1e9* CN |
| *KIR3DL1* CN + *KIR3DS1* CN = 2 |
| *KIR2DS3* CN + *KIR2DS5* CN = *KIR2DL5* CN |
| *KIR2DS1* CN = *KIR3DS1* CN |
| *KIR2DS5* CN = *KIR2DS1* CN |
| *KIR2DS4* (total) CN + *KIR2DS1* CN = 2 |
| *KIR2DS4FL* + *KIR2DS4Del* = *KIR2DS4* (total) CN |
| *KIR3DL2e4* CN = *KIR3DL2e9* CN |
| *KIR3DL2e4* CN + *KIR3DL2e9* CN = 4 |

**Supplementary Table 13: Checks to identify unexpected results in *KIR* copy number data.**

The tests check whether the copy number data for each sample conform to standard *KIR* haplotypes (haplotypes with frequency >1%) ([Jiang et al. 2012](#_ENREF_5)). CN = copy number result from the assay. *KIR2DS4* (total) refers to assay for the gene not the alleles (full-length variant [FL] and deletion variant [Del]).

| Gene | Primer | Sequence 5´ - 3´ | Primer size (-mer) | Exon | Approximate product size (bp) |
| --- | --- | --- | --- | --- | --- |
| *KIR2DL1* | Forward | TGGACCAAGAGTCTGCAGGA | 20 | 7 | 340 |
|  | Reverse | TGTTGTCTCCCTAGAAGACG | 20 | 9 |  |
| *KIR2DL2* | Forward | GAGGGGGAGGCCCATGA**G**T | 19 | 5 | 150 |
|  | Reverse | TCGAGTTTGACCACTCGT**G**T | 20 | 5 |  |
| *KIR2DL3* | Forward | CTTCATCGCTGGTGCTG | 17 | 7 | 550 |
|  | Reverse | AGGCTCTTGGTCCATTACAA | 20 | 8 |  |
| *KIR2DL5* | Forward | GGAGGACATGTGACTCTTCT | 20 | 3 | 200 |
|  | Reverse | GACCACTCAATGGGGGAGC | 19 | 3 |  |
| *KIR2DS1* | Forward | CTTCTCCATCAGTCGCATGAA | 21 | 4 | 100 |
|  | Reverse | AGGGTCACTGGGAGCTGACAA | 21 | 4 |  |
| *KIR2DS2* | Forward | CGGGCCCCACGGTTT | 15 | 5 | 240 |
|  | Reverse | GGTCACTCGAGTTTGACCACTCA | 23 | 5 |  |
| *KIR2DS3* | Forward | TGGCCCACCCAGGTCG | 16 | 4 | 240 |
|  | Reverse | TGAAAACTGATAGGGGGAGTGAGG | 24 | 4 |  |
| *KIR2DS4* | Forward | CTGGCCCTCCCAGGTCA | 17 | 4 | 200 |
|  | Reverse | TCTGTAGGTTCCTGCAAGGACAG | 23 | 4 |  |
| *KIR2DS4del* | Forward | CGGTTCAGGCAGGAGAGAAT | 20 | 5 | 250 |
|  | Reverse | TGACGGAAACAAGCAGTGGA | 20 | 5 |  |
| *KIR2DS5* | Forward | TCCAGAGGGTCACTGGGC | 18 | 4 | 210 |
|  | Reverse | AGAGAGGGGACGTTTAACC | 19 | 4 |  |
| *KIR3DL1* | Forward | CGCTGTGGTGCCTCGA | 16 | 3 | 200 |
|  | Reverse | GGTGTGAACCCCGACATG | 18 | 3 |  |
| *KIR3DS1* | Forward | AGCCTGCAGGGAACAGAAG | 19 | 8 | 300 |
|  | Reverse | GCCTGACTGTGGTGCTCG | 18 | 9 |  |

**Supplementary Table 14: The set of secondary assays for each gene.**

These reactions can be used to verify copy number results. The primary set of reactions (Supplementary Table 3) do not miss any known alleles or recombinants of *KIR* genes in populations of European-ancestry ([Gonzalez-Galarza et al. 2015](#_ENREF_4)). The primers and probes were carefully designed to avoid all known *KIR* gene polymorphism in their annealing sites. In Supplementary Tables 3 and 5 we list all the known alleles that could be missed in non-European populations.

In most cases, the listed ‘missed’ alleles have only been seen once i.e. there is only a single example and it has not been seen again. These likely represent sequencing artefacts or extremely rare alleles. For the others listed, the allele is only present in populations of African ancestry. We included two assays for *KIR3DL2* and *KIR3DL1*, which have known rare alleles in African populations. It would therefore be generally not necessary and inefficient to use the second set of assays when typing samples of European-ancestry, depending on the aim of the study.

For all the listed ‘missed’ alleles referred to above, the nucleotide substitution occurs in the middle of the primer or towards the 5´ end. This means that the PCR will still amplify the allele, albeit potentially less efficiently, and this will be detected by the real-time instrument. The result will still be accurate or will be flagged for further investigation because it does not fall as a discrete copy number (i.e. between integers). In these rare cases, the sample can be sequenced to verify the allele present, or the secondary assay can be used to verify the copy number.

If an assay is disrupted by a rare SNP (true allele dropout) this is identified by the loss of linkage with an adjacent gene that is known to be in high linkage disequilibrium; all loci have another locus in tight linkage or have an expected copy number e.g. framework genes are usually always two copies. One can check the data against predefined ‘standard *KIR* haplotype rules’ (Supplementary Table 13) to identify unexpected results and these samples can be further investigated. Alternatively, inconsistencies can be found using the KIR Haplotype Identifier on-line tool by the appearance of an unusual haplotype in the results.

Ninety-four per cent of haplotypes carry conventional *KIR* copy number in samples of European-ancestry ([Jiang et al. 2012](#_ENREF_5)). If the person carries a rare non-conventional haplotype, then usually more than one gene is duplicated or truncated. The incidence of one gene being miscalculated is extremely rare. For example, there was no discordance between the two reactions for *KIR3DL1* in the 1,698 samples.

The secondary *KIR2DS5* reaction does not amplify allele 2DS5*003 (~2% carrier frequency in African-origin populations; undetected elsewhere ([Gonzalez-Galarza et al. 2015](#_ENREF_4))). In combination, the primary and secondary assays do not miss any known alleles.

The nucleotides marked in bold in the *KIR2DL2* primer sequences are mismatches to the *KIR2DL2* annealing site as well as to other *KIR* gene sequences to improve specificity.

Forty unrelated samples, selected at random from the HBDI panel, were typed using replicate reactions (Roche Lightcycler 480) comprising DNA, primers, Taq polymerase, and buffer. Assays were carried out in singleplex using the *STAT6* as reference for relative quantification. The typing results showed complete concordance with the results from the primary assays (data not shown).

**REFERENCES**

Ashouri E, Ghaderi A, Reed EF, Rajalingam R. 2009. A novel duplex SSP-PCR typing method for KIR gene profiling. *Tissue Antigens* **74**: 62-67.

Bustin SA. 2004. *A-Z of quantitative PCR*. International University Line.

Degenhardt JD, de Candia P, Chabot A, Schwartz S, Henderson L, Ling B, Hunter M, Jiang Z, Palermo RE, Katze M et al. 2009. Copy number variation of CCL3-like genes affects rate of progression to simian-AIDS in Rhesus Macaques (Macaca mulatta). *PLoS Genet* **5**: e1000346.

Gonzalez-Galarza FF, Takeshita LY, Santos EJ, Kempson F, Maia MH, da Silva AL, Teles e Silva AL, Ghattaoraya GS, Alfirevic A, Jones AR et al. 2015. Allele frequency net 2015 update: new features for HLA epitopes, KIR and disease and HLA adverse drug reaction associations. *Nucleic Acids Res* **43**: D784-788.

Jiang W, Johnson C, Jayaraman J, Simecek N, Noble J, Moffatt MF, Cookson WO, Trowsdale J, Traherne JA. 2012. Copy number variation leads to considerable diversity for B but not A haplotypes of the human KIR genes encoding NK cell receptors. *Genome Res* **22**: 1845-1854.

Martin MP, Carrington M. 2008. KIR locus polymorphisms: genotyping and disease association analysis. *Methods in molecular biology (Clifton, NJ* **415**: 49-64.

Vilches C, Castano J, Gomez-Lozano N, Estefania E. 2007. Facilitation of KIR genotyping by a PCR-SSP method that amplifies short DNA fragments. *Tissue Antigens* **70**: 415-422.
